# Higher integration of the whole-brain resting state theta band network is associated with spatial working memory

**DOI:** 10.3389/fnhum.2026.1748183

**Published:** 2026-02-06

**Authors:** Anna Pavlova, Timofey Adamovich, Sergey Malykh

**Affiliations:** 1 Cognitive Health and Intelligence Center, Institute of Cognitive Neuroscience, HSE University, Moscow, Russia; 2 Developmental Behavioral Genetics Laboratory, Federal Research Centre of Psychological and Interdisciplinary Studies, Moscow, Russia

**Keywords:** brain network topology, EEG, functional connectivity, graph theory, resting state, spatial working memory, whole-brain connectivity

## Abstract

The aim of the present study was to investigate the association between resting-state EEG functional connectivity and spatial working memory (SWM) in adolescents, using a graph-theoretical approach. Sixty-three healthy adolescents (38 females; *M* = 16.8, SD = 0.47) participated in the study. Resting-state EEG was recorded by a wireless dry-sensor 32-channel headset (Cognionics Quick-32r) configured in a standard 10–20 montage. Graph metrics were calculated in alpha, theta, beta-low and beta-high frequency bands, using connection-strength thresholds of 50 and 80%. SWM performance was measured with the computerized Corsi Block-Tapping Task (CBT). Robust bootstrapped linear regressions revealed significant associations between graph metrics and CBT accuracy only for theta and beta-low frequency bands. Specifically, characteristic path length and modularity correlated negatively with CBT accuracy, while participation coefficient correlated positively, indicating that participants with more integrated resting-state networks performed better on the SWM task. A *k*-means clustering analysis divided the participants into two groups characterized by either integrated (low CPL and modularity, high participation coefficient) or segregated (high CPL and modularity, low participation coefficient) network organization. Participants with integrated theta-band networks demonstrated significantly higher CBT accuracy than those with segregated networks, whereas no such effect was observed in the beta band. Overall, theta-band networks were more integrated than beta-band networks. These findings highlight the importance of global integration of resting-state functional brain networks, particularly within the theta frequency range, for spatial working memory performance in adolescence.

## 1 Introduction

Working memory (WM) enables the temporary maintenance and manipulation of information to support reasoning, comprehension, and learning (Baddeley, 1992) while spatial working memory (SWM), a key component of WM, supports the short-term retention of spatial locations (Constantinidis and Wang, 2004). Converging neuroimaging and electrophysiological evidence shows that SWM relies on a distributed fronto-parietal regions with contributions from the hippocampus, thalamus, and cerebellum (Linden, 2007; Ashida et al., 2019; Constantinidis and Wang, 2004; Hampson et al., 2006; Shrager et al., 2008; Funahashi, 2013).

Because SWM depends on coordinated activity across multiple brain regions, a network-level view provides a powerful approach to understand the neural mechanisms behind differences in WM performance.

The graph-theoretical approach is widely applied to resting-state neuroimaging data to investigate how properties of a brain network (constructed from nodes, or brain regions, and edges, representing the functional connectivity or statistical interaction between their activity) are associated with various cognitive processes (Farahani et al., 2019). Recent studies have demonstrated that network properties derived from graph theory correlate with a variety of behavioral measures (He and Evans, 2010). Moreover, graph metrics of the human brain functional network show high reproducibility and can thus be considered relatively stable features of brain functional organization (Deuker et al., 2009).

From a graph-theoretical perspective, human brain networks are typically characterized by a so-called “small-world” topology, representing a balance between high local specialization (segregation) and high global integration, which ensures both low cost and high efficiency of information transmission (Sporns, 2013). While local specialization is critical for motor execution, working memory is predominantly associated with global network integration (Cohen and D’Esposito, 2016). This is explained by the complex interplay between modality-specific sensory areas and higher-order parietal, temporal, and frontal regions that support working memory processes (Linden, 2007; Palva et al., 2010; Rezayat et al., 2022). Several studies have shown that during working-memory tasks, local segregation decreases in favor of higher global integration (Toppi et al., 2018; Zippo et al., 2018). Similarly, Dai et al. (2017) found increased functional integration in the theta band and decreased segregation in the alpha band during working-memory tasks compared with control conditions.

Most existing evidence on the neural basis of working memory comes from task-based studies emphasizing dynamic reconfiguration of functional networks under cognitive load. However, the extent to which baseline (resting-state) network organization constrains working-memory performance remains unclear. Telling the difference between the natural structure of a brain network (i.e., trait effect) and how it changes when doing a task (i.e., state effect) helps us figure out if working memory ability is a stable trait or just a temporary state.

Because it lacks any purposeful activity, resting-state EEG is particularly well-suited for this task, as it provides a direct measure of baseline neural function without the confounding effects of a cognitive activity.

Despite accumulating evidence linking resting-state activity to working-memory performance (Hampson et al., 2006; Pashkov and Dakhtin, 2025), few studies have examined this relationship using graph-theoretical approaches in healthy populations. Most prior work has focused on clinical samples, where altered small-world properties in alpha and gamma bands have been associated with cognitive decline (Vecchio et al., 2016; Jouzizadeh et al., 2021). Thus, it remains unclear how resting-state network topology relates to SWM in typically developing adolescents.

Because resting-state EEG networks are organized in frequency-dependent ways, the relationship between network topology and behavior may vary across bands and connectivity thresholds. In particular, theta oscillations have consistently been implicated in WM control (Riddle et al., 2020; Sauseng et al., 2010; Hsieh and Ranganath, 2014), while beta rhythms have been linked to motor and maintenance processes (Engel and Fries, 2010). Thresholding also affects the results as strong or weak connections contribute differently to network measures and predictive power (Zakharov et al., 2020).

The present study investigates whether resting-state whole-brain network topology predicts spatial working-memory performance in adolescents. Specifically, we examine baseline network integration as a trait-like framework that supports subsequent task performance, without directly assessing task-induced network reconfiguration. We examined graph-theoretical properties across four frequency bands (theta, alpha, low-beta, and high-beta) and two connectivity thresholds (50 and 80%). Based on task-based evidence, we hypothesized that higher global integration—reflected in shorter characteristic path length, lower modularity, and higher participation coefficient—would be associated with superior SWM performance, particularly in the theta band at the 50% threshold. By situating these findings within the broader framework of baseline network integrity versus task-induced reconfiguration, this study contributes to understanding how intrinsic brain organization supports cognitive efficiency.

## 2 Materials and methods

### 2.1 Participants

Overall, 92 high school students studying in 10th grade participated in the study. However, we removed samples with low quality of EEG recording, as well as participants whose CBT score was 0 and those with reaction time higher than 30,000 mc. Final sample consists of 63 adolescents with age range from 16 to 17 years old (38 female, *M* = 16.8, SD = 0.47).

The study was approved by the Ethics Committee of the Psychological Institute of the Russian Academy of Education (protocol code 2020/4-1, date of approval 2 April 2020) in accordance with the Helsinki Declaration 2013. Informed consent was signed by participants of the study (as well as by their legal tutors in case of minor age).

### 2.2 Instruments

A computerized version of the Corsi Block-Tapping (CBT) task was administered to assess visuospatial working memory. The task included 12 trials (two sequences per level) with increasing difficulty ranging from 4 to 9 items. It followed a standard adaptive Corsi paradigm. At the beginning of each trial, nine blocks appeared in fixed positions on the screen. A subset of these blocks was sequentially highlighted in yellow, beginning with two items and increasing by one following each correct response. Each highlighted block was displayed for 300 ms, separated by a 300 ms inter-stimulus interval. Upon completion of the sequence, an auditory cue indicated the start of the response phase, during which participants reproduced the sequence by clicking the corresponding blocks with a computer mouse. Cursor movement was disabled during sequence presentation and enabled only for the response phase. Participants were required to complete their responses within 10 s; if no input occurred within that period, the task proceeded automatically to the next trial. Accuracy was computed by comparing each reproduced sequence to the target sequence. The task terminated when participants either correctly reproduced a sequence of nine items or made two consecutive errors. The primary performance measure was the CBT accuracy, defined as the longest sequence accurately recalled.

### 2.3 EEG data acquisition and preprocessing

Resting-state EEG data were acquired over a 10-min session using a wireless dry-sensor 32-channel headset (Cognionics Quick-32r) configured in a standard 10–20 montage. The choice of a portable EEG device was made due to the necessity to conduct the study in school settings. Participants were instructed to maintain a relaxed state, minimize movement, and avoid falling asleep. The session consisted of alternating two-minute blocks of eyes-open and eyes-closed conditions, cued by verbal instructions (“Now open your eyes,” “Now close your eyes”). Only the data recorded during the eyes-closed condition were used for the present analysis.

Preprocessing was performed using the MNE-Python and autoreject software packages. Continuous EEG data was processed using a comprehensive, multi-stage pipeline designed for robust artifact suppression. Initial preparation included identifying and removing non-physiological channels, interpolating bad channels, and re-referencing to the Common Average Reference (CAR). Data were then band-pass filtered (1–40 Hz), divided by condition (eyes open/closed), and segmented into one-second epochs with linear detrending. Artifact handling began with a coarse epoch rejection based on a peak-to-peak amplitude threshold (> 125 mkV). Subsequently, the data-driven sensor-repair algorithm, RANSAC, was applied to the remaining epochs to detect and correct problematic sensors. The final step involved applying the automated epoch-cleaning algorithm, AutoReject, for definitive data cleaning. Data cleaning resulted in the removal of approximately 40% of epochs and the interpolation of 3 channels per recording.

Functional connectivity estimation was conducted on the cleaned EEG epochs using the frites package, with data handling performed via MNE-Python and leveraging CUDA acceleration for computational efficiency. The core metric employed was Gaussian-copula mutual information (GCMI), calculated to assess the non-linear functional coupling between all sensor pairs. GCMI has been increasingly used in neuroimaging data analysis (Ince et al., 2017), including EEG time-series data (Thuraisingham, 2018). Unlike coherence, PLV, or wPLI, which primarily quantify linear dependencies or phase-locked interactions, GCMI is sensitive to both linear and non-linear dependencies between signals, making it well suited for resting EEG where interactions are not necessarily phase-consistent or stimulus-locked. Importantly, the copula-based formulation renders GCMI invariant to the marginal distributions of the signals, providing robustness to the non-Gaussian amplitude distributions commonly observed in EEG data. At the same time, as a sensor-level connectivity measure, GCMI remains sensitive to volume conduction and spatial mixing effects (Ashrafi and Soltanian-Zadeh, 2022). In a prior study using the same GCMI-based connectivity framework, we directly quantified the impact of spatial proximity and common sources by comparing MI between neighboring and non-neighboring electrodes (Adamovich et al., 2024; Supplementary Material 1). While such effects were present, they were relatively small and did not obscure network-level organization. Also, zero-lag interactions are sensitive to volume conduction, converging evidence indicates that zero- or near-zero-lag synchronization is a genuine and functionally relevant feature of large-scale brain networks. Empirical and theoretical work shows that such interactions can reflect meaningful neural coordination and network integration, and their wholesale removal may distort large-scale connectivity structure (Mehra et al., 2025; Gollo et al., 2014; Palva and Palva, 2018).

This analysis was performed iteratively across four distinct frequency bands: Theta (4–8 Hz), Alpha (8–13 Hz), Low Beta (13–20 Hz), and High Beta (20–30 Hz). For each band, epochs were first band-pass filtered to isolate the specific range. The connectivity metric was calculated across all channels for every clean epoch, and the resulting connectivity matrices were subsequently averaged across all available epochs to yield a single, time-aggregated functional connectivity estimate per subject and per frequency band.

After computing the epoch-wise functional connectivity matrices for each subject and frequency band using Gaussian-copula mutual information, each raw matrix was thresholded at quantile levels of 0.5 (actual network density was approximately 50%) and 0.8 (actual network density was approximately 20%) to create sparse weighted undirected graphs. Thresholds of 0.5 and 0.8 were chosen to probe network topology under both a relatively inclusive regime (50% strongest connections retained) as we did in our previous work (Zakharov et al., 2020) and a more conservative regime emphasizing only the strongest functional connections (top 20%). In contrast to fixed absolute thresholds, quantile-based thresholding controls for inter-individual variability in overall connectivity magnitude. Minimum spanning tree approaches, while advantageous for guaranteeing full network connectedness, were not adopted here because they impose a highly constrained topology and may discard physiologically meaningful medium-strength connections that are relevant for large-scale integration measures.

Using the igraph Python library, the following graph metrics were computed at each threshold:

*Characteristic path length***—**median of the shortest-path distances across all connected node pairs. Characteristic path length was computed on fully connected graphs; network construction enforced global connectivity, such that disconnected nodes and undefined path lengths did not occur.

*Clustering coefficient*—a measure of the number of edges between a node’s nearest neighbors or the fraction of triangles around a node, and is a measure of functional segregation. Mean clustering coefficient was used in the analysis.

*Eigenvector centrality*—a measure of the influence of a node in a network. A high eigenvector score means that a node is connected to many nodes that themselves have high scores. Mean eigenvector centrality was used in the analysis.

*Betweenness centrality*—quantifies a node’s influence in a network by measuring how often it lies on the shortest paths between pairs of other nodes, reflecting its potential to control or monitor information flow. Mean betweenness centrality was used in the analysis.

*Participation coefficient*—a measure of a node’s connectivity diversity across network communities, quantifying how evenly its edges are distributed among different modules in complex networks. Was computed via the Leiden algorithm. Mean participation coefficient was used in the analysis.

*Modularity*—a measure of functional segregation, which quantifies how well the network can be subdivided into non-overlapping groups of nodes or modules. Was computed via the Leiden algorithm.

*Mean rich-club coefficient*—a measure describes a network structure where highly connected nodes (the “rich” nodes with many links) form an interconnected core, exhibiting denser connections among themselves than expected by random chance. Rich-club coefficient curve was generated, and its mean summary statistic was reported.

### 2.4 Statistical analysis

All analyses were conducted in Python 3.14. Descriptive statistics and distribution checks were performed for all variables included in the analysis. To reduce the influence of extreme values, robust statistical methods were applied.

To examine the relationship between graph-theoretical metrics and SWM performance, robust linear regression with bootstrapping (10,000 iterations) was used. In each model, a single graph metric (characteristic path length, modularity, clustering coefficient, or participation coefficient) served as a predictor, while CBT accuracy or mean reaction time were dependent variables. The variables included in the model were z-standardized. Bootstrapped 95% confidence intervals (CI) were computed to assess the stability of the estimates. Additionally, bootstrapped Spearman correlation coefficients were calculated to provide non-parametric measures of association less sensitive to outliers and non-normality.

To examine multicollinearity and mutual dependencies between graph metrics, pairwise Spearman correlations were computed separately for each frequency band and each connection-strength threshold (50 and 80%).

To divide samples on those with relatively segregated and integrated brain networks, k-means clustering was applied to three key metrics—characteristic path length, modularity, and participation coefficient. All the variables were z-standardized before clustering. *K* = 2 was chosen as the best option due to both high interpretability (i.e., 2 contrast clusters) and the highest drop in distortion metric for *k* = 2 (see Supplementary Figure 3.1). Importantly, the clustering solution for *k* = 2 was stable across multiple random initializations (*n* = 10), yielding highly consistent cluster assignments. To assess the robustness and significance of the identified network subtypes, we conducted two complementary analyses. First, a resampling-based stability analysis was performed using bootstrap resampling (*n* = 1,000 iterations, sample fraction = 80%), with the Adjusted Rand Index (ARI) used to quantify the consistency of the clustering solution across resampled datasets. Second, a permutation-based silhouette test was applied to evaluate whether the observed cluster structure deviates from the null hypothesis of a single homogeneous population. For this test, each feature (network metric) was independently shuffled across subjects in 1000 permutations to destroy inter-feature correlations, and K-means clustering (*k* = 2) was applied to each permuted dataset. The silhouette score was computed for each permutation, generating a null distribution against which the observed silhouette score was compared. The proportion of null permutations with silhouette scores equal to or exceeding the observed value was reported as the permutation-based *p*-value, providing a formal test of cluster significance.

Between-cluster differences in CBT accuracy were tested using non-parametric Manna-Whitney test and confirmed with robust linear regression.

## 3 Results

Descriptive statistics and distributions of all variables included in the analysis are provided in the Supplementary materials (Supplementary Tables 1.1–1.3; Supplementary Figures 1.1–1.5).

Significant associations between graph metrics and CBT accuracy were observed only for the theta and beta-low frequency bands. Table 1 reports bootstrapped robust linear regression coefficients for models where each graph metric served as the only predictor and CBT accuracy was the dependent variable. Graph metrics were computed at two thresholds: 50 and 80%.

**Table 1:** Bootstrapped robust linear regression coefficients for beta and theta band connectivity metrics and CBT accuracy.

Graph theory metrics	Beta low		Theta	
	50%	80%	50%	80%
CPL	−0.38^**^ [−0.58; −0.13]	−0.30^*^ [−0.55; −0.06]	−0.37^**^ [−0.60; −0.20]	−0.32^**^ [−0.53; −0.12]
Participation index	0.29^*^ [0.04; 0.58]	0.26^*^ [0.01; 0.50]	0.39^**^ [0.20; 0.59]	0.26^*^ [0.02; 0.47]
Modularity	−0.28^*^ [−0.47; −0.01]	*−0.23* [−0.45; 0.07]	−0.25^*^ [−0.43; −0.02]	−0.24^*^ [−0.45; −0.03]
Eigenvector centrality	−0.06 [−0.32; 0.15]	−0.09 [−0.37; 0.13]	0.04 [−0.21; 0.29]	−0.02 [−0.29; 0.28]
Betweenness centrality	−0.07 [−0.31; 0.09]	0.06 [−0.20; 0.31]	0.01 [−0.30; 0.29]	0.02 [−0.26; 0.29]
Cluster coefficient	−0.1 [−0.35; 0.15]	0.2 [−0.07; 0.39]	−0.29^*^ [−0.52; −0.07]	−0.01 [−0.30; 0.27]
Rich club	−0.08 [−0.29; 0.14]	0.18 [−0.01; 0.57]	−0.06 [−0.59; 0.03]	−0.10 [−0.08; 0.43]

^*^p-value < 0.05, ^**^p-value < 0.001, [] – 95% CI, CPL, Characteristic Path Length, *N* = 63. Near-significant effects are in italics.

Overall, characteristic path length (CPL) and modularity were negatively associated with CBT accuracy, showing moderate effect sizes across both frequency bands and thresholds. In contrast, the participation coefficient correlated positively with CBT accuracy under all conditions. The clustering coefficient showed a weak negative correlation with CBT accuracy only in the theta band at the 50% threshold.

Correlations between graph metrics and CBT accuracy are illustrated in Supplementary Figures 2.1–2.4. Bootstrapped Spearman correlation coefficients are presented in Supplementary Table 2.1.

No significant associations were found between graph metrics and mean reaction time. The corresponding regression coefficients, correlation coefficients, and scatterplots are presented in Supplementary Tables 2.2–2.3 and Supplementary Figures 2.5–2.8, respectively.

Because CPL, modularity, and participation coefficient showed the most robust associations with CBT accuracy, subsequent analyses focused on these metrics. These measures were strongly intercorrelated (*r* = 0.66–0.84) across thresholds and frequency bands: CPL and modularity were positively correlated with each other, while participation coefficient was negatively correlated with both (see Supplementary Tables 2.4–2.6).

To further explore network-level differences, we applied k-means clustering to divide participants into two groups: participants with low modularity and CPL but high participation coefficient (i.e., more integrated networks), and participants with high modularity and CPL but low participation coefficient (i.e., more segregated networks).

Clustering was performed separately for theta and beta bands at both thresholds (50 and 80%). For the 50% threshold, the mean ARI was 0.79 (theta) and 0.76 (beta), with median ARI of 0.92 (theta) and 0.78 (beta). For the 80% threshold, the mean ARI was 0.67 (theta) and 0.73 (beta), with median ARI of 0.78 (theta) and 0.92 (beta). Across all four conditions, clusters were highly stable and yielded statistical significance in permutation-based silhouette tests against a single-population null model (*p*-value < 0.001), indicating that the observed network subtypes reliably reflect distinct patterns rather than sampling noise.

Across all conditions, the clusters were well differentiated in the metrics used for clustering. However, clustering based on the beta band yielded higher quality: mean silhouette coefficients for clustering using the beta band were 0.42 and 0.43, and for the theta band—0.40 and 0.31; distortion—86 and 89 for the beta band and 99 and 116 for the theta band for the thresholds 50 and 80% correspondingly (see Supplementary Figures 3.1–3.4 and Supplementary Table 3.1 for more details).

**Figure 1:**
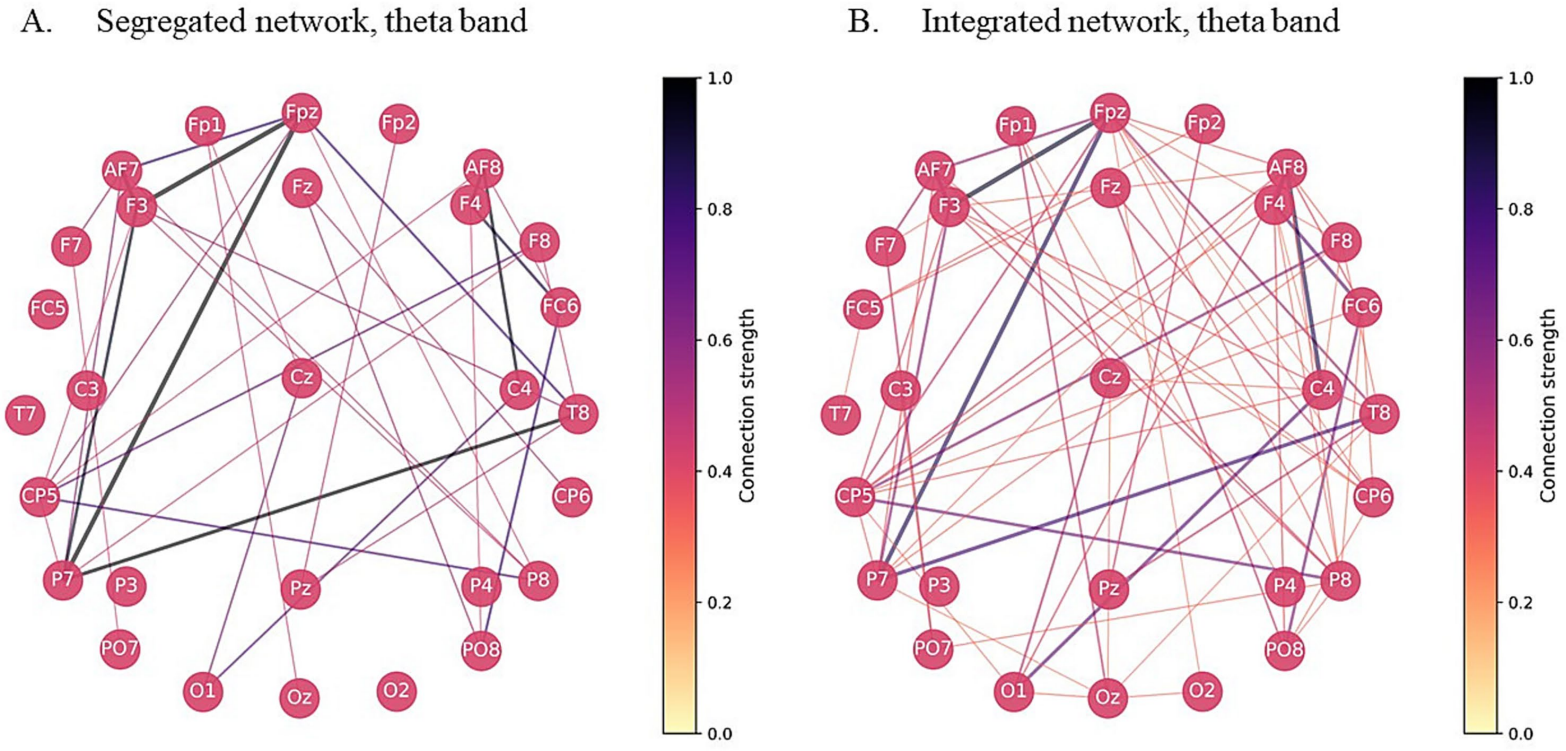
The averaged brain network topology for children with segregated **(A)** and integrated **(B)** brain networks, theta band. The darker color indicates higher connectivity strength. Clusters obtained in 50% threshold were used for visualization purposes. For the group with segregated brain network M (CPL) = 1.38, M (Participation index) = 0.85, M (Modularity) = 0.00; for the group with integrated brain network M (CPL) = 1.00, M (Participation index) = 0.88, M (Modularity) = −0.02.

Notably, clustering results differed across frequency bands: nearly one-third of participants belonged to the segregated cluster in one frequency band but to the integrated cluster in the other (see Supplementary Tables 4.1–4.2). Therefore, clustering results for theta and beta bands were treated as relatively independent variables. Figure 1 illustrates theta-band network topology for the integrated and segregated clusters; the corresponding beta-band networks are shown in Supplementary Figure 3.5.

Between-cluster comparisons of CBT accuracy are presented in Table 2. Despite higher clustering quality in the beta band, no significant differences in CBT accuracy were observed between clusters in this frequency. In contrast, for the theta band, participants with integrated networks demonstrated significantly higher CBT accuracy than those with segregated networks at both thresholds (Supplementary Figure 4.1).

**Table 2:** Between-cluster differences in CBT score.

Frequency band, threshold	Integrated network M (SD)	Segregated network M (SD)	Mean difference	Manna–Whitney *U* stats	*p*-value
Beta band, 50%	6.45 (2.24)	5.5 (2.04)	0.95	630.5	*0.06*
Theta band, 50%	6.66 (1.98)	4.83 (2.06)	1.83	696.0	0.001**
Beta band, 80%	6.36 (2.27)	5.64 (2.08)	0.70	591.5	0.17
Theta band, 80%	6.56 (2.04)	5.0 (2.06)	1.56	660.0	0.006**

Near-significant effects are in italics; ^*^p-value < 0.05, ^**^p-value < 0.001, *N* = 63.

Robust linear regression including clustering results for both frequency bands confirmed that brain network structure predicts CBT accuracy for the theta band but not for the beta band (Table 3). For the 80% threshold, the theta-band effect reached marginal significance. No interaction between theta and beta clustering was observed. These results suggest that when theta-band network organization is taken into account, beta-band architecture no longer contributes to the prediction of SWM performance (Supplementary Figure 4.2). Gender was not controlled in the analysis, as no gender difference was observed for CBT accuracy (Manna–Whitney *U* = 539.0, *p*-value = 0.36).

**Table 3:** Robust linear regression results.

Variables	*B*	Standard error	Beta	*p*-value	95% CI
Threshold 50%					
Intercept	7.09	0.43	16.62	<0.001**	[6.25; 7.93]
Segregated network theta	−1.89	0.90	−2.11	0.035*	[−3.65; −0.13]
Segregated network beta	−0.82	0.69	−1.19	0.234	[−2.17; 0.53]
Segregated network theta * Segregated network beta	0.05	1.16	0.04	0.966	[−2.23; 2.33]
Threshold 80%					
Intercept	6.95	0.48	14.46	<0.001**	[6.01; 7.89]
Segregated network theta	−1.75	0.98	−1.79	0.07	[−3.67; 0.17]
Segregated network beta	−0.66	0.73	−0.91	0.37	[−2.09; 0.77]
Segregated network theta * Segregated network beta	0.23	1.25	0.18	0.85	[−2.22; 2.67]

^*^p-value < 0.05, ^**^p-value < 0.001, *N* = 63.

Comparisons of CPL, modularity, and participation coefficient between beta and theta bands revealed that theta-band networks are generally more integrated than beta-band networks—that is, they show significantly lower CPL and modularity and higher participation coefficient (Supplementary Figure 5, Supplementary Tables 5.1–5.2).

## 4 Discussion

This study examined whether intrinsic, resting-state functional network organization predicts spatial working-memory (SWM) performance in adolescents. This question addresses a central issue in cognitive neuroscience: the relative contribution of baseline network integrity (trait-like properties of brain organization) versus task-related reconfiguration (state-dependent dynamics) to individual differences in cognitive ability. Our findings indicate that greater resting-state integration (i.e., lower characteristic path length and modularity, higher participation coefficient), particularly in the theta band, is associated with superior SWM performance—suggesting that stable intrinsic connectivity patterns may reflect a neural framework associated with more efficient working-memory processing. Although task-induced reconfiguration was not directly assessed in the present study, these results are consistent with the view that intrinsic network topology acts as a permissive or enabling constraint on subsequent cognitive processing.

Characteristic path length is computed as the median of the shortest-path distances across all connected node pairs, providing a measure of how efficiently information or signals can propagate through the network (Noschese and Reichel, 2024). A low characteristic path length is typically observed in highly integrated networks, reflecting high global efficiency (Fronczak et al., 2004). The negative association between characteristic path length and SWM performance aligns with the neural efficiency hypothesis, which posits that individuals with higher cognitive abilities exhibit lower and more efficient brain activation (Neubauer and Fink, 2009). Clinical studies also report negative associations between characteristic path length and cognitive performance in patients with systemic lupus erythematosus (Li et al., 2024) and carotid stenosis (Chang et al., 2016). Langer et al. (2012) similarly found a negative association between characteristic path length and non-verbal intelligence. However, some studies have reported contradictory findings; for instance, Zakharov et al. (2020) found a positive association between characteristic path length and non-verbal intelligence.

Modularity quantifies the density of within-community connections relative to a random network and reflects the degree of segregation in the network (Baum et al., 2017). Previous studies have shown that modularity is negatively associated with cognitive performance in patients with carotid stenosis (Chang et al., 2016) and is increased (particularly in the theta and delta bands) in patients with Alzheimer’s disease compared with healthy controls (Gits, 2016). However, other studies have reported positive associations between modularity and executive functioning (Baum et al., 2017) or working memory (Stevens et al., 2012) in healthy populations.

The participation coefficient reflects the diversity of a brain region’s connections and serves as an index of global integration (Sporns, 2018). Previous research has shown that a higher participation coefficient predicts more effective transfer of working memory training to other cognitive domains (Chen et al., 2022).

As characteristic path length, modularity, and participation coefficient were all highly intercorrelated, we were able to classify participants into two groups: those with more integrated networks (low characteristic path length and modularity, high participation coefficient) and those with more segregated networks (high characteristic path length and modularity, low participation coefficient). As expected, participants with more integrated networks demonstrated higher SWM performance. This finding is consistent with previous work emphasizing the importance of global integration for working memory processes (Cohen and D’Esposito, 2016; Toppi et al., 2018; Zippo et al., 2018; Dai et al., 2017) and support the relevance of a baseline network integrity for working memory performance. Previously, stronger network integration in resting state was linked to enhanced general cognitive ability in healthy adults (Wang et al., 2021). Moreover, functional connectivity between posterior cingulate cortex, medial frontal gyrus and ventral anterior cingulate cortex was associated with working memory performance both during task performance and at rest (Hampson et al., 2006).

These results extend the neural efficiency hypothesis (Neubauer and Fink, 2009) by suggesting that individuals with more globally integrated resting-state networks may require less dynamic reconfiguration when engaging in WM tasks. In this sense, intrinsic network efficiency could represent a candidate trait-like property associated with more flexible engagement of distributed regions under cognitive load.

From a developmental perspective, adolescence marks a critical period of increasing long-range integration and decreasing local segregation in large-scale brain networks (Baum et al., 2017). The association between brain network integrity and SWM performance observed here may reflect this maturational trajectory, whereby more globally efficient intrinsic networks support the emergence of higher executive functions. Importantly, the present study did not explicitly model age-related effects, as the sample was highly homogeneous with respect to age (the majority of participants were 16 years old), limiting meaningful within-sample variability. We would expect similar associations between resting-state network integration and working memory performance in adults, consistent with the neural efficiency hypothesis discussed above. In contrast, such relationships may be less pronounced or qualitatively different in younger children, where large-scale network integration is still emerging and cognitive performance may rely more strongly on compensatory or locally segregated processing.

Notably, network integration in the theta band showed a more robust association with SWM performance than did integration in the beta band. Moreover, the beta-band effect disappeared when controlling for the theta-band effect. The functional relevance of theta oscillations for working memory is well established (Pomper and Ansorge, 2021). Theta activity has been identified as a key mechanism for coordinating hippocampal and prefrontal networks during working memory tasks (Tesche and Karhu, 2000; Raghavachari et al., 2001). Resting-state theta activity has also been linked to task-related theta power during conflict monitoring (Pscherer et al., 2020) and motor inhibition (Pscherer et al., 2021, 2022). Overall, resting-state theta-band oscillations are thought to provide a baseline that modulates how the brain activates under cognitive demand (Zou et al., 2012).

The dominance of the theta band in predicting SWM performance also aligns with its well-established role in coordinating prefrontal-hippocampal communication and fronto-parietal synchrony important for encoding (Soltani Zangbar et al., 2020; Su et al., 2024; Jacob et al., 2018). In contrast, the weaker and less stable effects in the beta band likely reflect more local or sensorimotor-related dynamics that are less directly relevant to SWM. Theta band also plays important role in cognitive control, an essential component of working memory (Badre and Nee, 2018).

Interestingly, the theta-band network was characterized by higher integration compared with the beta-band network, consistent with previous findings during visual working memory maintenance (Palva et al., 2010). This supports the notion that the theta band plays a pivotal role in coordinating distributed brain networks (Prochnow et al., 2022).

Importantly, although effect sizes varied across thresholds, the direction and presence of the main associations—particularly in the theta band—were consistent across both network densities. This convergence across thresholds increases confidence that the observed effects are not driven by a specific threshold choice but reflect stable properties of baseline network organization. The stronger predictive power of graph metrics at the 50% threshold suggests that networks comprising both strong and weak connections better capture individual variability in cognitive efficiency. This finding underscores the importance of preserving weak but functionally meaningful links when quantifying whole-brain connectivity, consistent with the notion that distributed coordination depends on a balance between strong local and weak long-range ties.

Several limitations should be considered. First of all, the study employed a cross-sectional, correlational design, with resting-state EEG and spatial working-memory performance assessed at a single time point. Consequently, the observed associations between resting-state *θ*-band network integration and SWM performance cannot be interpreted in causal terms. Future studies combining longitudinal designs, cognitive training or intervention paradigms, and joint modeling of resting-state and task-evoked neural dynamics will be necessary to directly test whether higher baseline integration reduces the need for task-related reconfiguration and supports more efficient neural processing during working-memory demands. Secondly, sample size was relatively small, and graph metrics contained outliers that might have influenced the statistical results. Robust linear regression was applied to mitigate this issue. Although effect sizes are moderate, their consistency across multiple integration-related metrics and analytic approaches supports the robustness of the findings. Meanwhile, studies with larger samples are needed to replicate these findings. Additionally, all connectivity and graph-theoretical analyses were conducted at the sensor level, and Gaussian-copula mutual information, like most zero-lag–sensitive measures, may be influenced by volume conduction and field spread. Such effects can inflate instantaneous dependencies between nearby electrodes and may increase apparent global connectivity and integration metrics. Consequently, the present findings should be interpreted as reflecting large-scale statistical coordination patterns rather than direct neural interactions between distinct cortical sources. Finally, we did not control for drowsiness state that can markedly affect resting state theta-activity. However, we believe that regular switching between eyes-open and eyes-close states during EEG data recording prevents adolescents for drowsiness in majority of cases.

In summary, our findings demonstrate that spatial working-memory performance in adolescence is linked to the intrinsic integration of resting-state brain networks, particularly within the theta frequency range. This supports the idea that a well-integrated baseline architecture constitutes a neural predisposition for efficient working-memory processing. Future multimodal studies combining resting-state and task-based EEG or fMRI could clarify how baseline connectivity constrains the flexibility of network reconfiguration during cognitive engagement.

## Data Availability

The raw data supporting the conclusions of this article will be made available by the authors, without undue reservation.
