# Supplementary material for: Higher integration of the whole-brain resting state theta band network is associated with spatial working memory
Source: Front Hum Neurosci. 2026 Feb 6;20:1748183. doi: 10.3389/fnhum.2026.1748183 (PMC12920549; doi:10.3389/fnhum.2026.1748183)
Supplement: Supplementary file 1 [file Data_Sheet_1.docx]

Supplementary Material

# Descriptive statistics and visualization of the distribution of the variables included in the analysis

# Supplementary Table 1.1. Descriptive statistics for the behavioral variables

|  | Mean (SD) | Median | Range | Skewness | Kurtosis |
| --- | --- | --- | --- | --- | --- |
| CBT accuracy | 5.97 (2.18) | 6.0 | 2 - 10 | -0.03 | -0.83 |
| CBT mean reaction time | 6807.22 (7092.10) | 5242.0 | 2577 - 13272 | 1.96 | 5.08 |

*Note.* N = 63

# Supplementary Table 1.2. Descriptive statistics for the graph metrices, threshold 50%

|  | Mean (SD) | Median | Range | Skewness | Kurtosis |
| --- | --- | --- | --- | --- | --- |
| CPL, beta | 1.54 (0.46) | 1.49 | 0.42 – 2.8 | 0.35 | 0.16 |
| Participation index, beta | 0.76 (0.14) | 0.81 | 0.30 – 0.90 | -1.52 | 2.00 |
| Modularity, beta | 0.02 (0.03) | 0.01 | -0.04 – 0.09 | 0.13 | -0.58 |
| Eigenvector centrality, beta | 0.48 (0.04) | 0.48 | 0.38 – 0.55 | -0.36 | -0.31 |
| Betweenness centrality, beta | 12.01 (2.00) | 11.72 | 9.03 – 19.31 | 1.03 | 1.80 |
| Cluster coefficient, beta | 0.73 (0.03) | 0.73 | 0.66 – 0.78 | -0.08 | -0.89 |
| Rich club, beta | 0.72 (0.06) | 0.72 | 0.55 – 0.85 | -0.41 | 0.15 |
| CPL, theta | 1.15 (0.28) | 1.12 | 0.37 – 1.96 | 0.06 | 0.99 |
| Participation index, theta | 0.87 (0.03) | 0.87 | 0.79 – 0.92 | -0.78 | 0.81 |
| Modularity, theta | -0.01 (0.02) | -0.01 | -0.04 – 0.06 | 1.11 | 2.36 |
| Eigenvector centrality, theta | 0.46 (0.04) | 0.46 | 0.32 – 0.58 | -0.22 | 0.19 |
| Betweenness centrality, theta | 10.78 (8.10) | 10.48 | 8.10 – 15.34 | 0.61 | -0.47 |
| Cluster coefficient, theta | 0.69 (0.03) | 0.69 | 0.60 – 0.79 | 0.53 | 1.47 |
| Rich club, theta | 0.67 (0.05) | 0.68 | 0.50 – 0.78 | -0.56 | 0.64 |

*Note.* N = 63

# Supplementary Table 1.3. Descriptive statistics for the graph metrices, threshold 80%

|  | Mean (SD) | Median | Range | Skewness | Kurtosis |
| --- | --- | --- | --- | --- | --- |
| CPL, beta | 2.36 (0.67) | 2.29 | 0.66 – 4.02 | 0.26 | 0.16 |
| Participation index, beta | 0.50 (0.14) | 0.52 | 0.16 – 0.74 | -0.47 | -0.53 |
| Modularity, beta | 0.05 (0.06) | 0.03 | -0.05 – 0.19 | 0.30 | -0.80 |
| Eigenvector centrality, beta | 0.35 (0.03) | 0.35 | 0.27 – 0.41 | -0.15 | -0.67 |
| Betweenness centrality, beta | 26.88 (3.44) | 27.45 | 19.66 – 35.07 | 0.04 | -0.54 |
| Cluster coefficient, beta | 0.47 (0.04) | 0.47 | 0.39 – 0.57 | 0.28 | -0.49 |
| Rich club, beta | 0.58 (0.09) | 0.57 | 0.36 – 0.79 | 0.06 | -0.38 |
| CPL, theta | 1.92 (0.51) | 1.92 | 0.58 – 3.04 | -0.15 | 0.17 |
| Participation index, theta | 0.62 (0.06) | 0.63 | 0.52 – 0.74 | 0.09 | -0.71 |
| Modularity, theta, theta | 0.00 (0.04) | 0.00 | -0.06 – 0.14 | 1.31 | 2.84 |
| Eigenvector centrality, theta | 0.33 (0.04) | 0.33 | 0.24 – 0.42 | -0.14 | -0.42 |
| Betweenness centrality, theta | 25.72 (3.35) | 25.83 | 18.83 – 33.83 | 0.02 | -0.23 |
| Cluster coefficient, theta | 0.46 (0.05) | 0.45 | 0.37 – 0.56 | 0.16 | -0.71 |
| Rich club, theta | 0.55 (0.09) | 0.56 | 0.29 – 0.71 | -0.64 | 0.27 |

*Note.* N = 63


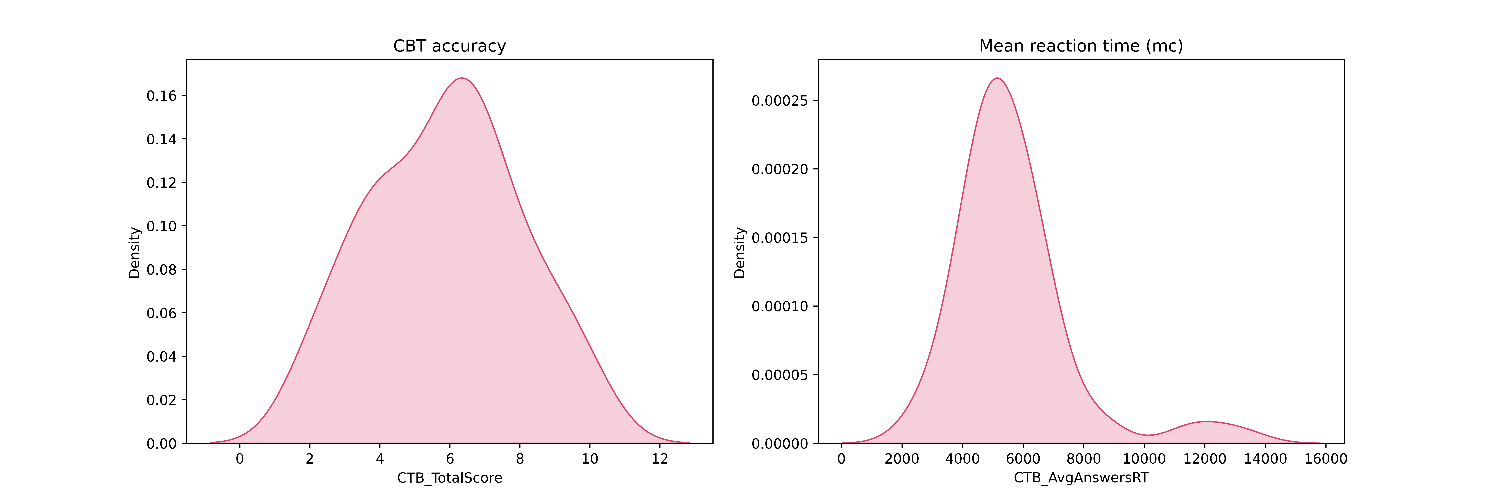


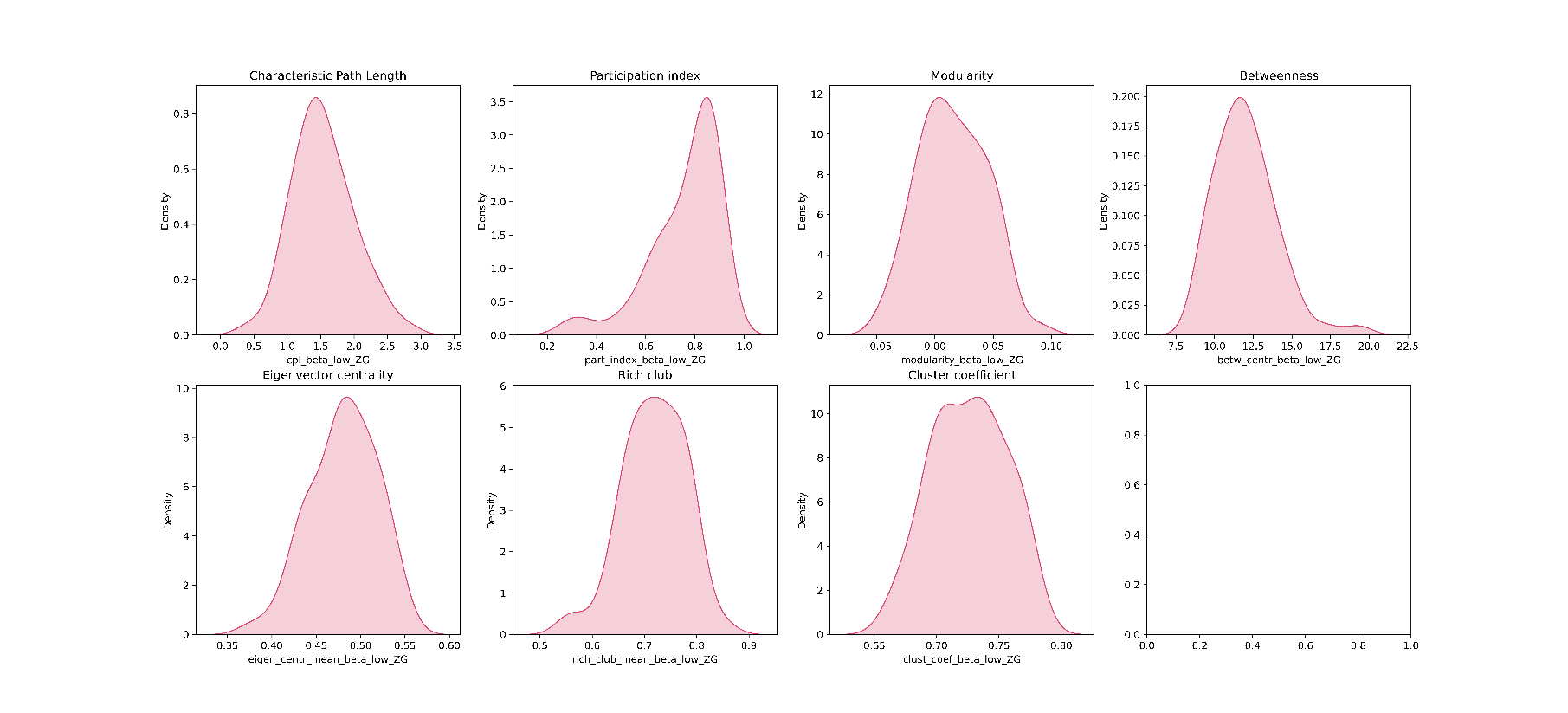
 **Supplementary Figure 1.1.** The distribution of the behavioral variables. The left panel depicts density plot for CBT accuracy (the highest span, a participant performs with no errors), the right panel depicts the density plot for mean reaction time (in milliseconds)

**Supplementary Figure 1.2.** The distribution of the graph metrics. Beta band, threshold 50%


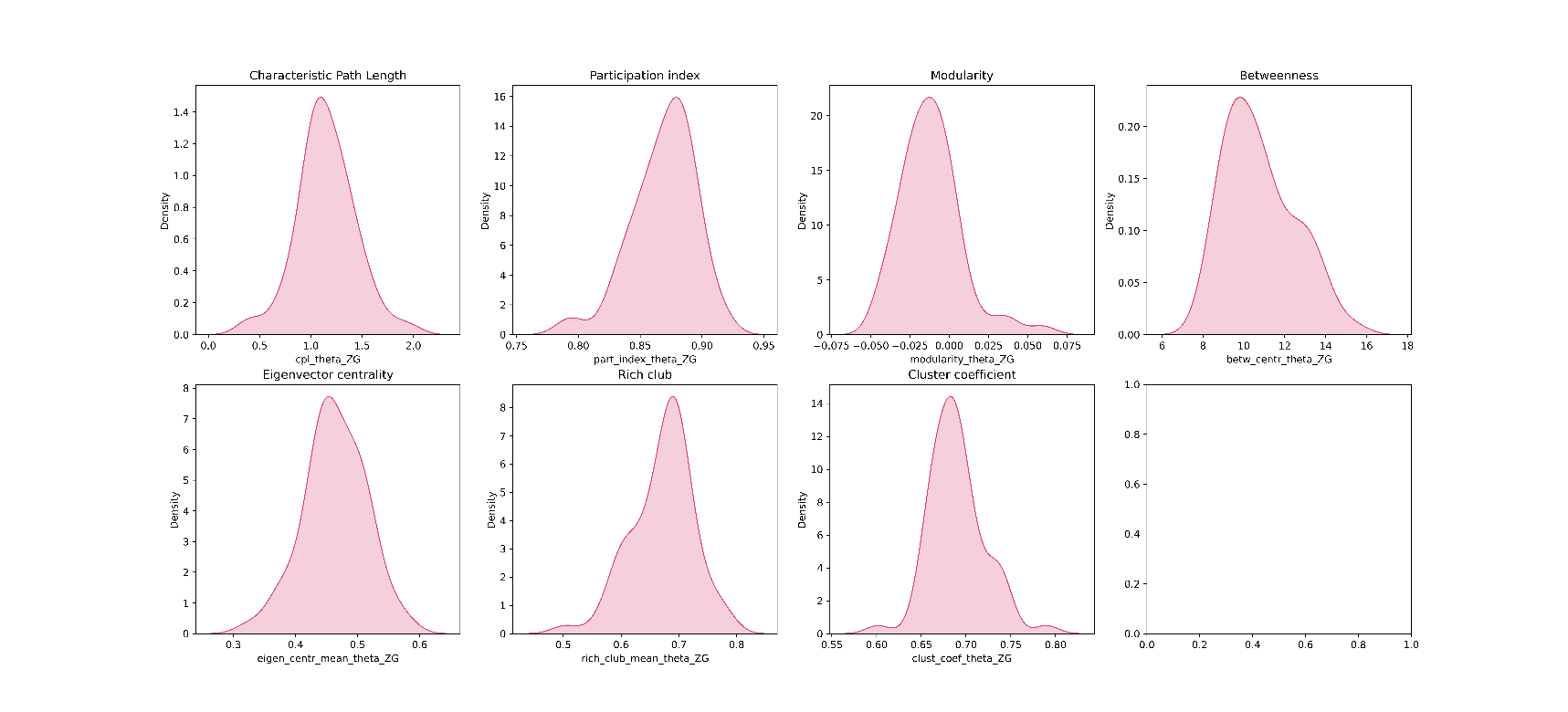


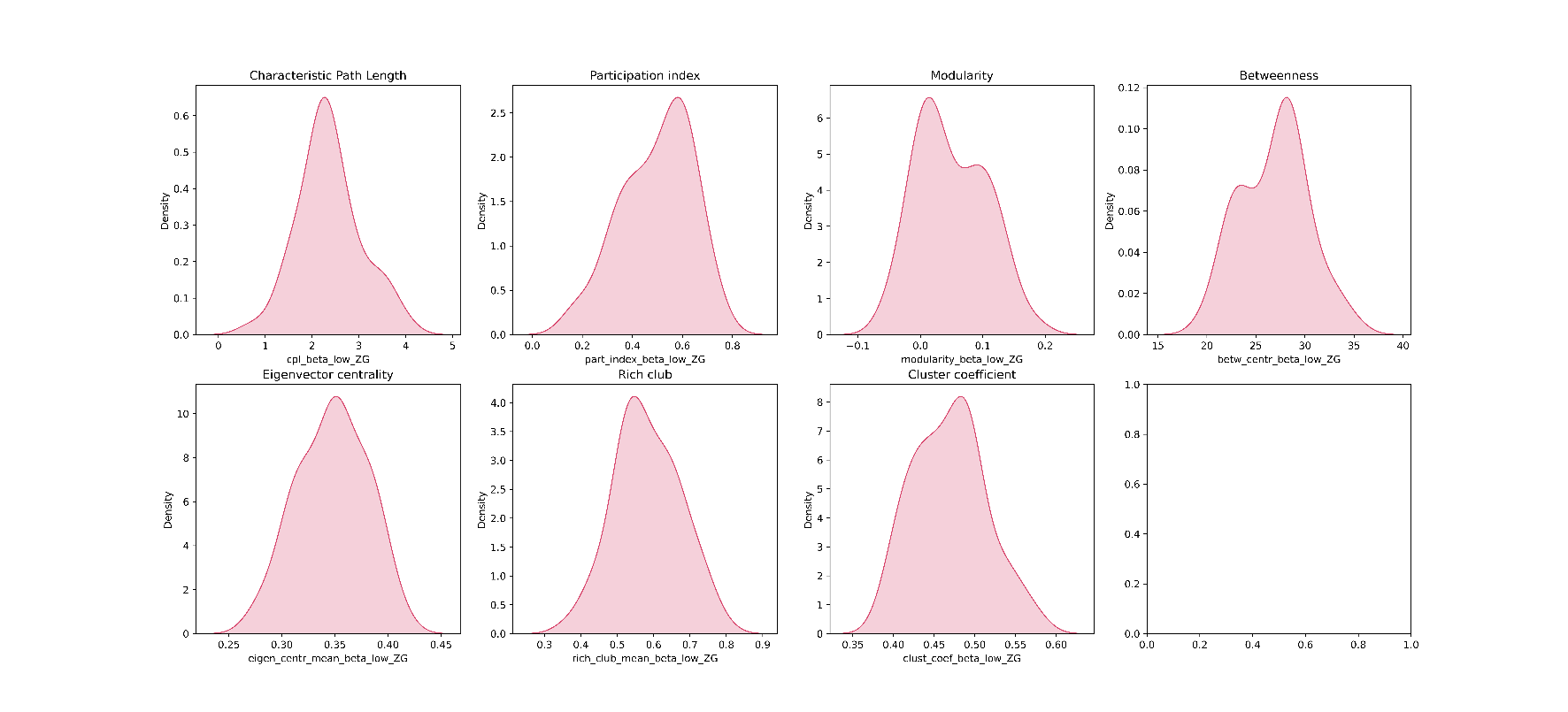
**Supplementary Figure 1.3.** The distribution of the graph metrics. Theta band, threshold 50%

**Supplementary Figure 1.4.** The distribution of the graph metrics. Beta band, threshold 80%

#
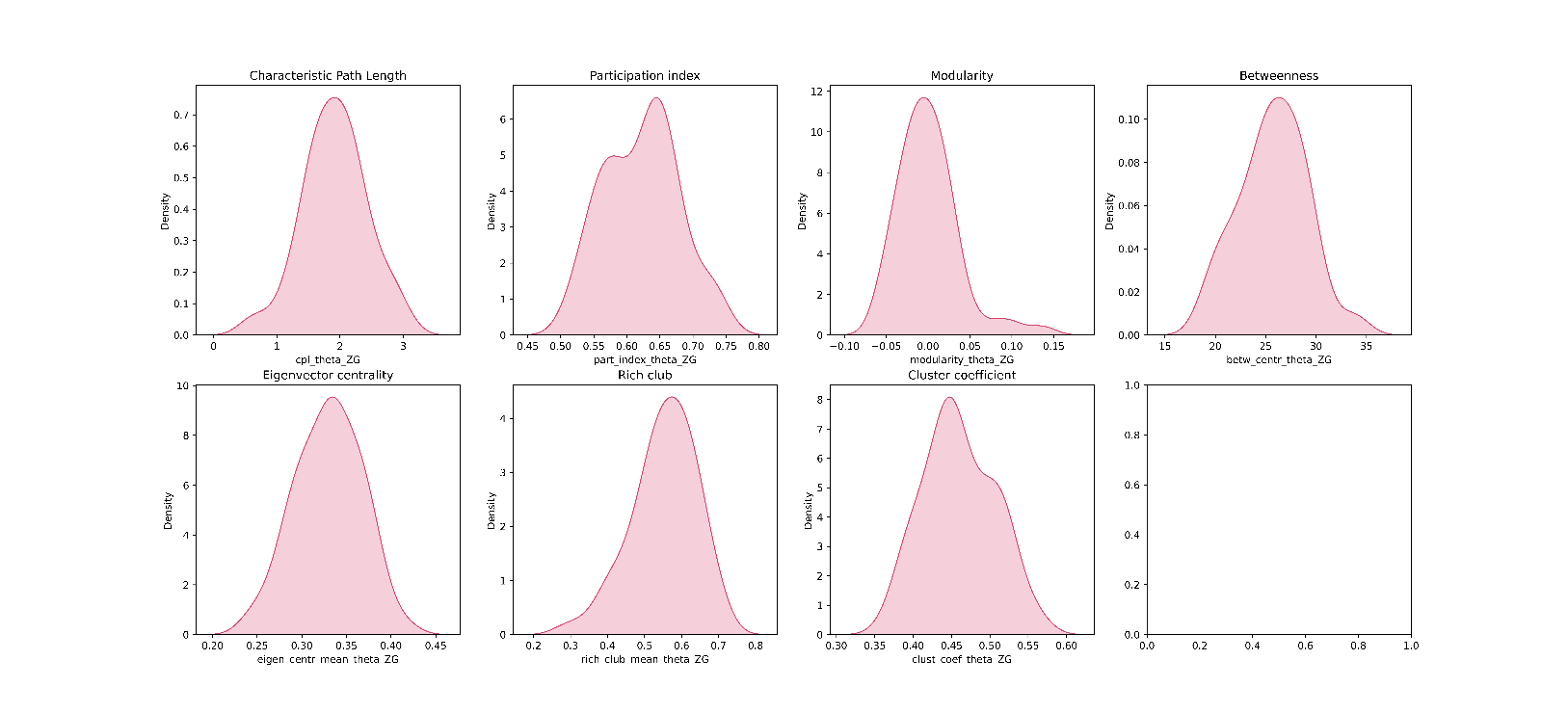


**Supplementary Figure 1.5.** The distribution of the graph metrics. Theta band, threshold 80%

# The associations between graph metrics and behavioral measures

# Supplementary Table 2.1. Bootstrapped spearman correlations beta and theta band and connectivity metrics and CBT accuracy

|  | Beta low | | Theta | |
| --- | --- | --- | --- | --- |
|  | 0.5 | 0.8 | 0.5 | 0.8 |
| CPL | -0.33*  [-0.57; -0.09] | -0.28*  [-0.54; -0.03] | -0.38*  [-0.60; -0.11] | -0.34*  [-0.56; -0.08] |
| Participation index | *0.21*  [-0.02; 0.45] | *0.17*  [-0.09; 0.42] | 0.35*  [0.13; 0.55] | *0.18*  [-0.05; 0.42] |
| Modularity | *-0.23*  [-0.47; 0.04] | *-0.20*  [-0.46; 0.07] | -0.26*  [-0.51; -0.02] | *-0.24*  [-0.48; 0.01] |
| Eigenvector centrality | -0.01  [-0.24; 0.22] | -0.03  [-0.28; 0.20] | 0.09  [-0.18; 0.35] | 0.07  [-0.20; 0.38] |
| Betweenness centrality | -0.11  [-0.33; 0.12] | 0.02  [-0.22; 0.27] | -0.03  [-0.31; 0.27] | -0.04  [-0.27; 0.21] |
| Cluster coefficient | *-0.19* [-0.43; 0.06] | 0.10 [-0.13; 0.33] | -0.29* [-0.51; -0.07] | 0.03  [-0.24; 0.30] |
| Rich club | -0.09 [-0.32; 0.14] | 0.14 [-0.12; 0.40] | -0.32* [-0.53; -0.08] | 0.08  [-0.13; 0.32] |

*Note.* *p-value < 0.05. N = 63

# Supplementary Table 2.2. Bootstrapped spearman correlations between beta and theta band and connectivity metrics and CBT reaction time

|  | Beta low | | Theta | |
| --- | --- | --- | --- | --- |
|  | 50% | 80% | 50% | 80% |
| CPL | -0.07  [-0.31; 0.18] | -0.05  [-0.30; 0.20] | -0.04  [-0.31; 0.21] | -0.01  [-0.27; 0.23] |
| Participation index | 0.04  [-0.23; 0.30] | 0.07  [-0.21; 0.34] | -0.02  [-0.29; 0.24] | -0.11  [-0.37; 0.16] |
| Modularity | -0.12  [-0.36; 0.14] | -0.10  [-0.33; 0.15] | 0.06  [-0.20; 0.32] | 0.03  [-0.22; 0.31] |
| Eigenvector centrality | -0.11  [-0.36; 0.16] | -0.02  [-0.28; 0.24] | -0.06  [-0.30; 0.18] | -0.06  [-0.31; 0.21] |
| Betweenness centrality | 0.01  [-0.26; 0.24] | -0.12  [-0.37; 0.15] | -0.04  [-0.29; 0.24] | 0.09  [-0.16; 0.34] |
| Cluster coefficient | 0.06  [-0.21; 0.33] | -0.03  [-0.27; 0.24] | 0.09  [-0.17; 0.37] | -0.17  [-0.43; 0.09] |
| Rich club | 0.07  [-0.17; 0.31] | 0.10  [-0.15; 0.34] | 0.03  [-0.26; 0.33] | 0.08  [-0.21; 0.37] |

*Note.* *p-value < 0.05. N = 63

# Supplementary Table 2.3. Bootstrapped robust linear regression coefficients for beta and theta band and connectivity metrics and CBT reaction time

|  | Beta low | | Theta | |
| --- | --- | --- | --- | --- |
|  | 50% | 80% | 50% | 80% |
| CPL | - 1. (0.84)   [-0.13; 0.13] | 0.02 (0.78)  [-0.13; 0.13] | - 1. (0.74)   [-0.12; 0.13] | 0.03 (0.71)  [-0.11; 0.13] |
| Participation index | -0.01 (0.93)  [-0.15; 0.18] | 0.01 (0.87)  [-0.13; 0.19] | -0.03 (0.67)  [-0.19; 0.13] | -0.08 (0.24)  [-0.23; 0.05] |
| Modularity | -0.06 (0.38)  [-0.19; 0.09] | -0.04 (0.55)  [-0.18; 0.12] | - 1. (0.90)   [-0.09; 0.14] | 0.00 (0.97)  [-0.10; 0.17] |
| Eigenvector centrality | -0.01 (0.84)  [-0.17; 0.12] | 0.03 (0.66)  [-0.10; 0.17] | -0.04 (0.61)  [-0.14; 0.09] | -0.02 (0.81)  [-0.15; 0.13] |
| Betweenness centrality | -0.02 (0.76)  [-0.16; 0.11] | -0.07 (0.29)  [-0.24; 0.08] | -0.05 (0.51)  [-0.21; 0.14] | 0.07 (0.34)  [-0.07; 0.21] |
| Cluster coefficient | - 1. (0.59)   [-0.15; 0.18] | -0.05 (0.47)  [-0.19; 0.07] | 0.06 (0.37)  [-0.11; 0.23] | -0.09 (0.21)  [-0.27; 0.06] |
| Rich club | - 1. (0.68)   [-0.12; 0.13] | 0.00 (0.98)  [-0.17; 0.14] | 0.07 (0.34)  [-0.18; 0.23] | 0.03 (0.73)  [-0.25; 0.22] |

*Note.* *p-value < 0.05. N = 63


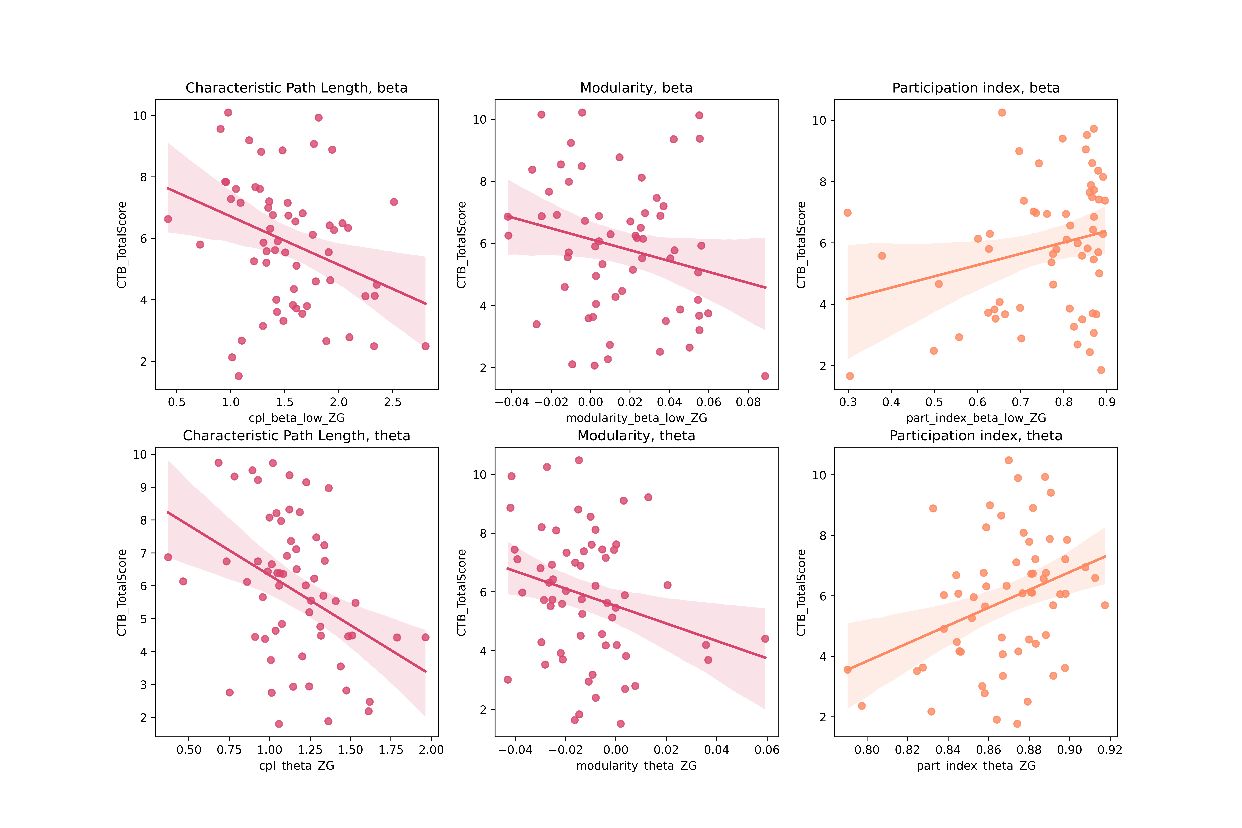


**Supplementary Figure 2.1.** The scatterplots for the relationship between CBT accuracy and CPL, modularity, participation index, threshold 50%. The top panel depicts scatterplot for the beta band, the bottom panel depicts scatterplots for the theta band


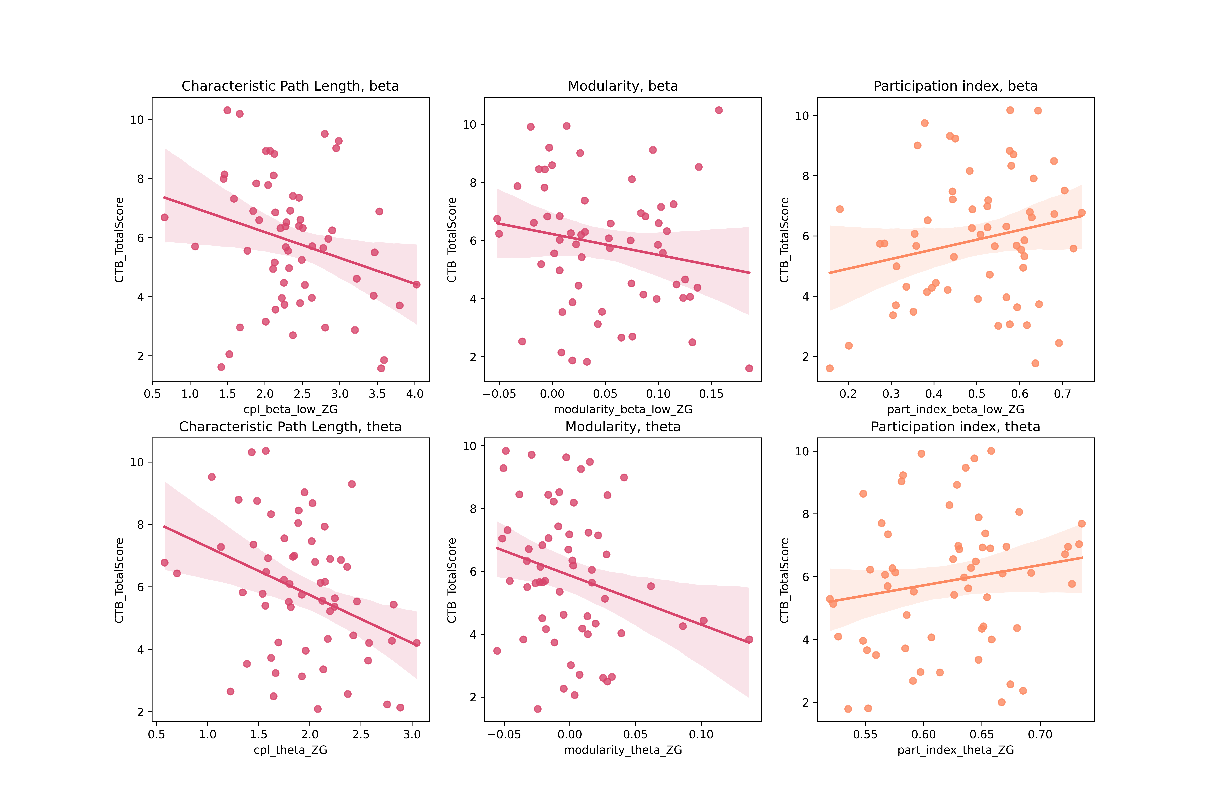


**Supplementary Figure 2.2.** The scatterplots for the relationship between CBT accuracy and CPL, modularity, participation index, threshold 80%. The top panel depicts scatterplot for the beta band, the bottom panel depicts scatterplots for the theta band


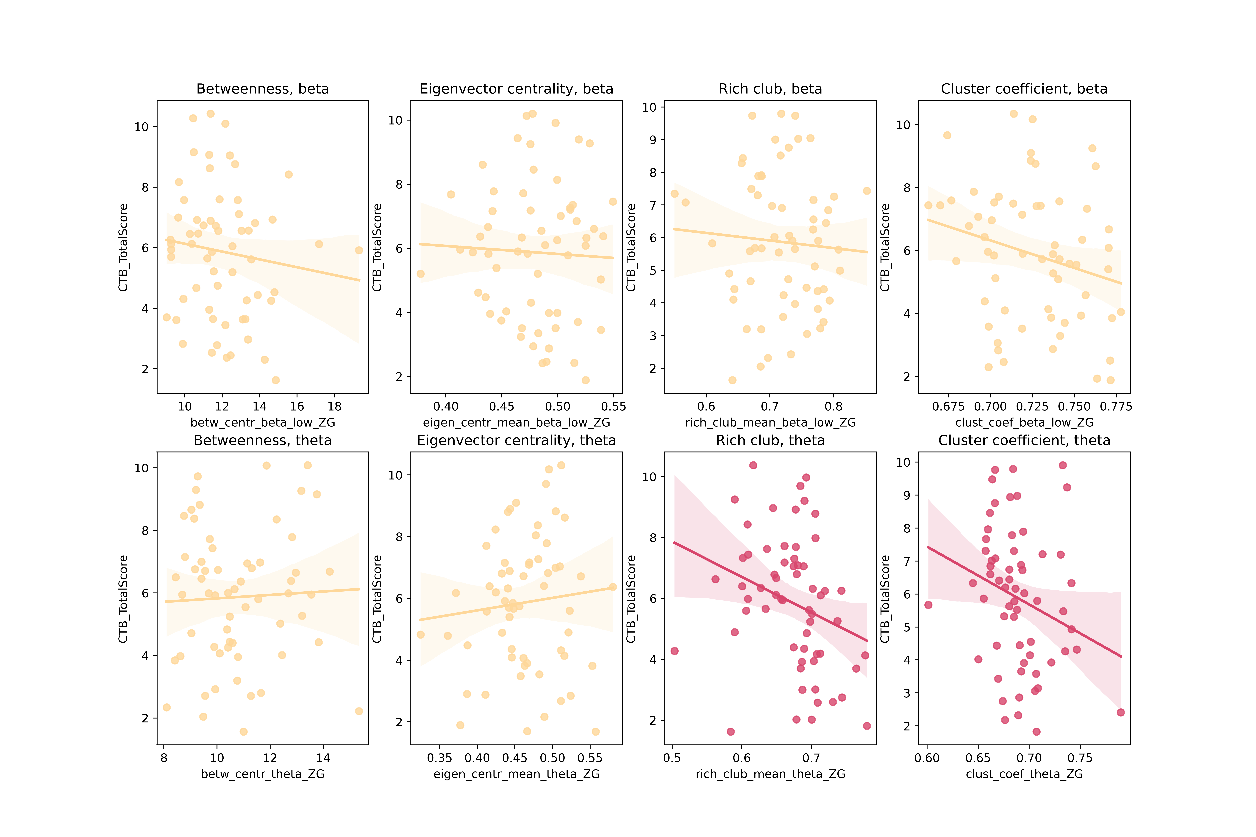


**Supplementary Figure 2.3.** The scatterplots for the relationship between CBT accuracy and other metrics, threshold 50%. The top panel depicts scatterplot for the beta band, the bottom panel depicts scatterplots for the theta band


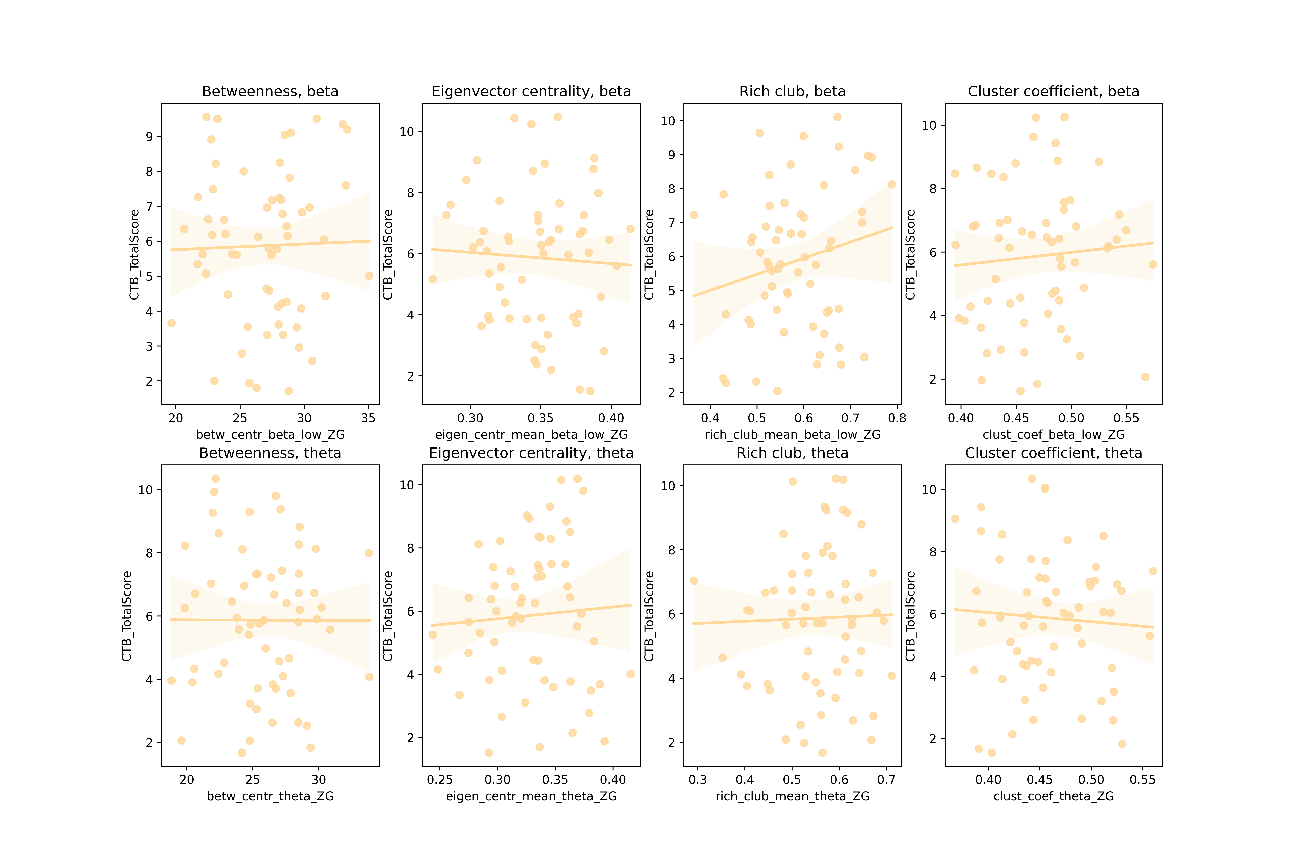


**Supplementary Figure 2.4.** The scatterplots for the relationship between CBT accuracy and other metrics, threshold 80%. The top panel depicts scatterplot for the beta band, the bottom panel depicts scatterplots for the theta band


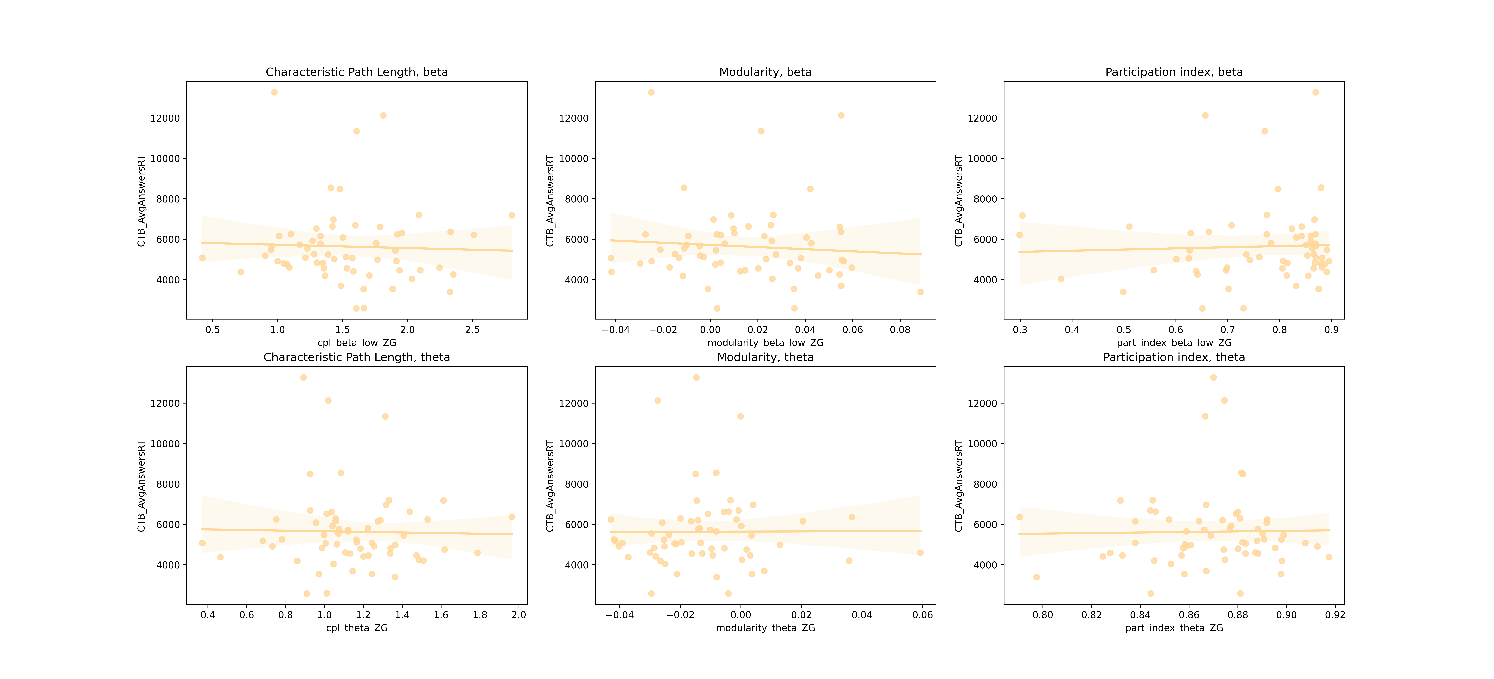


**Supplementary Figure 2.5.** The scatterplots for the relationship between CBT mean reaction time and CPL, modularity, participation index, threshold 50%. The top panel depicts scatterplot for the beta band, the bottom panel depicts scatterplots for the theta band


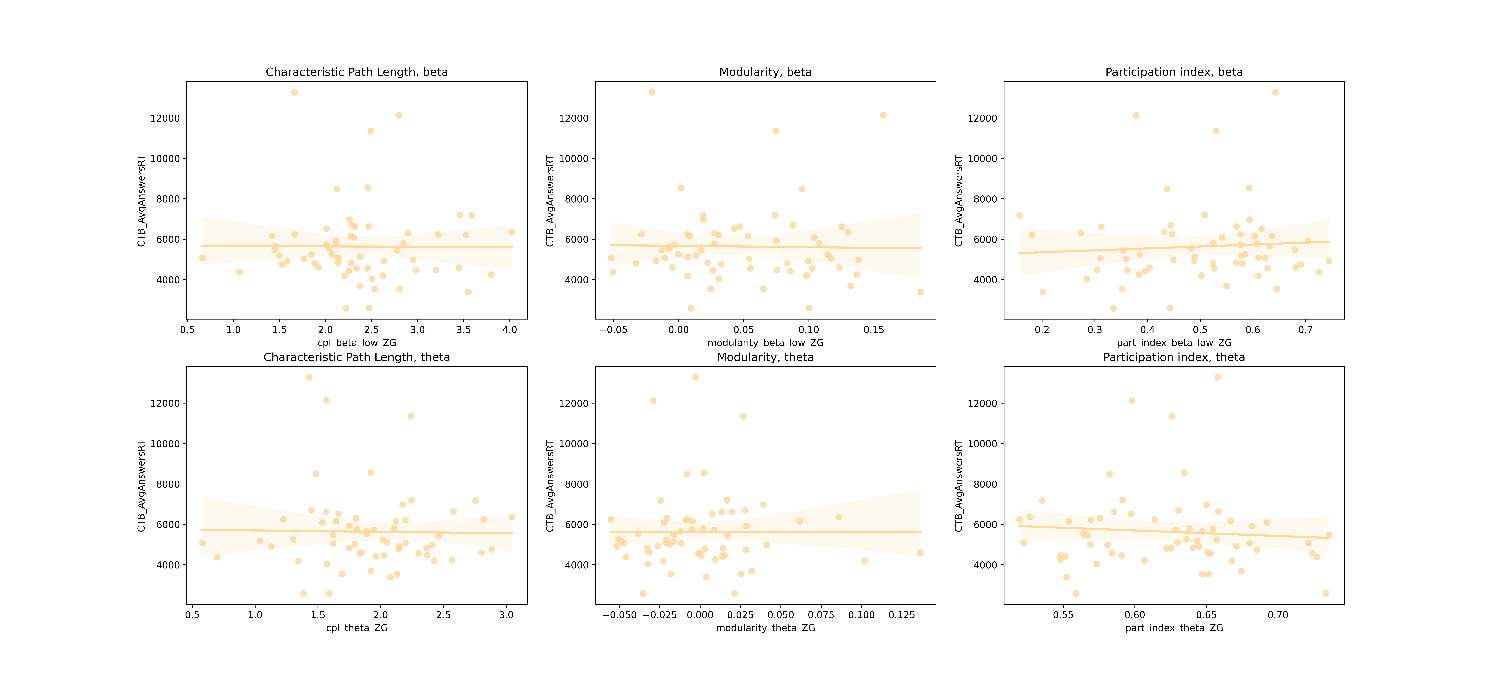


**Supplementary Figure 2.6.** The scatterplots for the relationship between CBT mean reaction time and CPL, modularity, participation index, threshold 80%. The top panel depicts scatterplot for the beta band, the bottom panel depicts scatterplots for the theta band


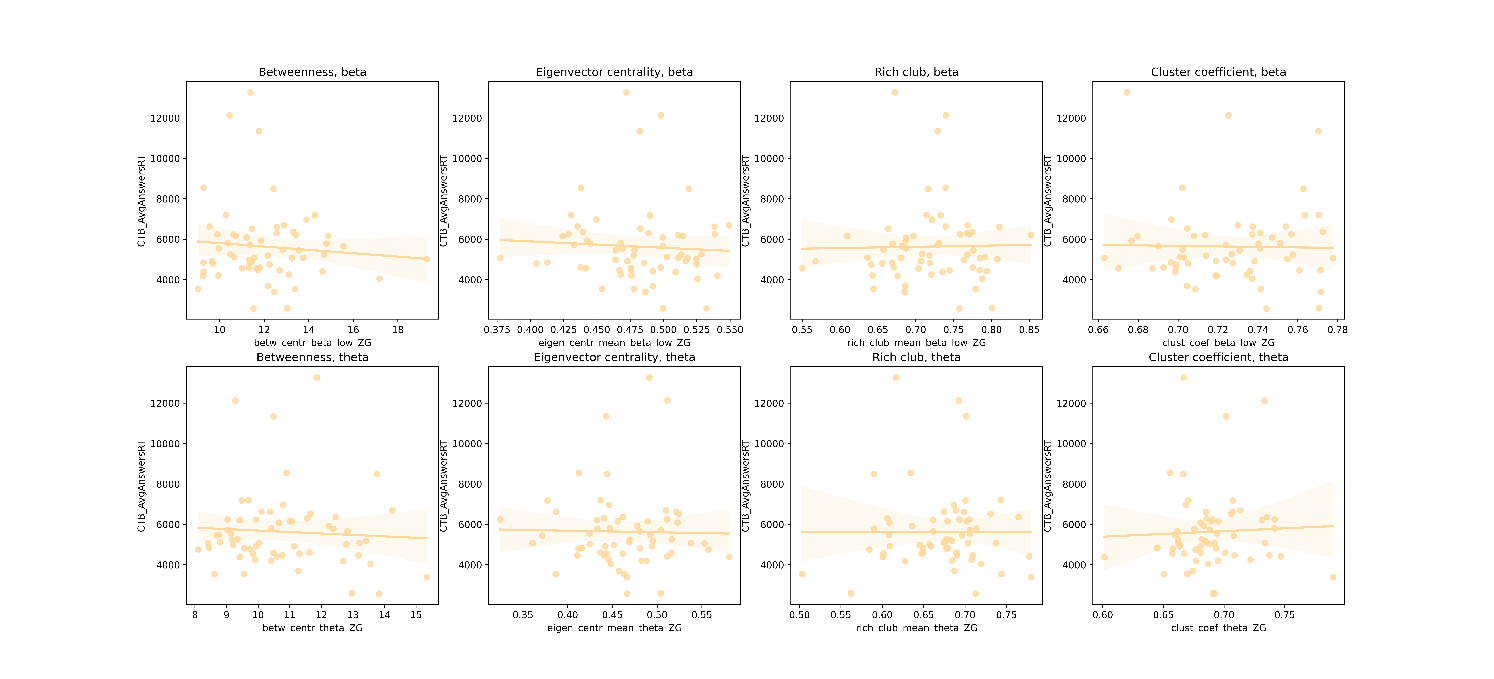


**Supplementary Figure 2.7.** The scatterplots for the relationship between CBT mean reaction time and other metrics, threshold 50%. The top panel depicts scatterplot for the beta band, the bottom panel depicts scatterplots for the theta band


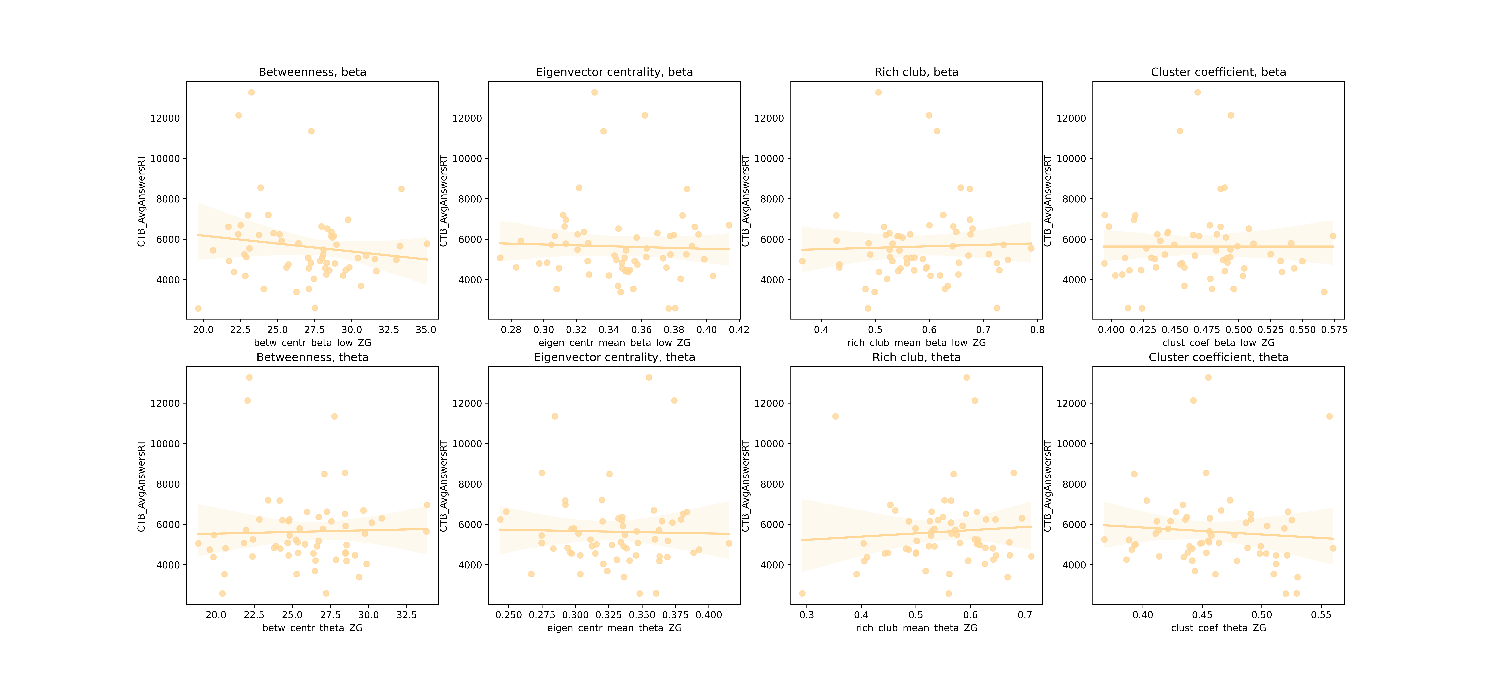


**Supplementary Figure 2.8.** The scatterplots for the relationship between CBT mean reaction time and other metrics, threshold 80%. The top panel depicts scatterplot for the beta band, the bottom panel depicts scatterplots for the theta band

# Supplementary Table 2.4. Spearman correlations between CPL, modularity and participation coefficient for beta and theta frequency bands, threshold 50%

|  | CPL beta | CPL theta | Modularity beta | Modularity theta | Participation index beta | Participation index theta |
| --- | --- | --- | --- | --- | --- | --- |
| CPL beta | 1 |  |  |  |  |  |
| CPL theta | 0.55** | 1 |  |  |  |  |
| Modularity beta | 0.71** | 0.44** | 1 |  |  |  |
| Modularity theta | 0.42** | 0.72** | 0.47** | 1 |  |  |
| Participation index beta | -0.84** | -0.35** | -0.67** | -0.25* | 1 |  |
| Participation index theta | 0.53** | -0.65** | -0.43** | -0.61** | 0.51** | 1 |

*Note.* *p-value < 0.05; **p-value < 0.01. N = 63

# Supplementary Table 2.5. Spearman correlations between CPL, modularity and participation coefficient for beta and theta frequency bands, threshold 80%

|  | CPL theta | CPL beta | Modularity theta | Modularity beta | Participation index theta | Participation index beta |
| --- | --- | --- | --- | --- | --- | --- |
| CPL theta | 1 |  |  |  |  |  |
| CPL beta | 0.59** | 1 |  |  |  |  |
| Modularity theta | 0.58** | 0.32** | 1 |  |  |  |
| Modularity beta | 0.40** | 0.60** | 0.52** | 1 |  |  |
| Participation index theta | -0.66** | -0.27* | -0.52** | -0.11 | 1 |  |
| Participation index beta | -0.51** | -0.42** | -0.14 | -0.12 | 0.60** | 1 |

*Note.* *p-value < 0.05; **p-value < 0.01. N = 63

# Supplementary Table 2.6. Spearman correlations between same metrices on thresholds 50% and 80%

|  | CPL | Modularity | Participation index |
| --- | --- | --- | --- |
| Theta | 0.98** | 0.93** | 0.63** |
| Beta | 0.94** | 0.96** | 0.94** |

*Note.* *p-value < 0.05; **p-value < 0.01. N = 63

# Clustering quality and between-cluster differences


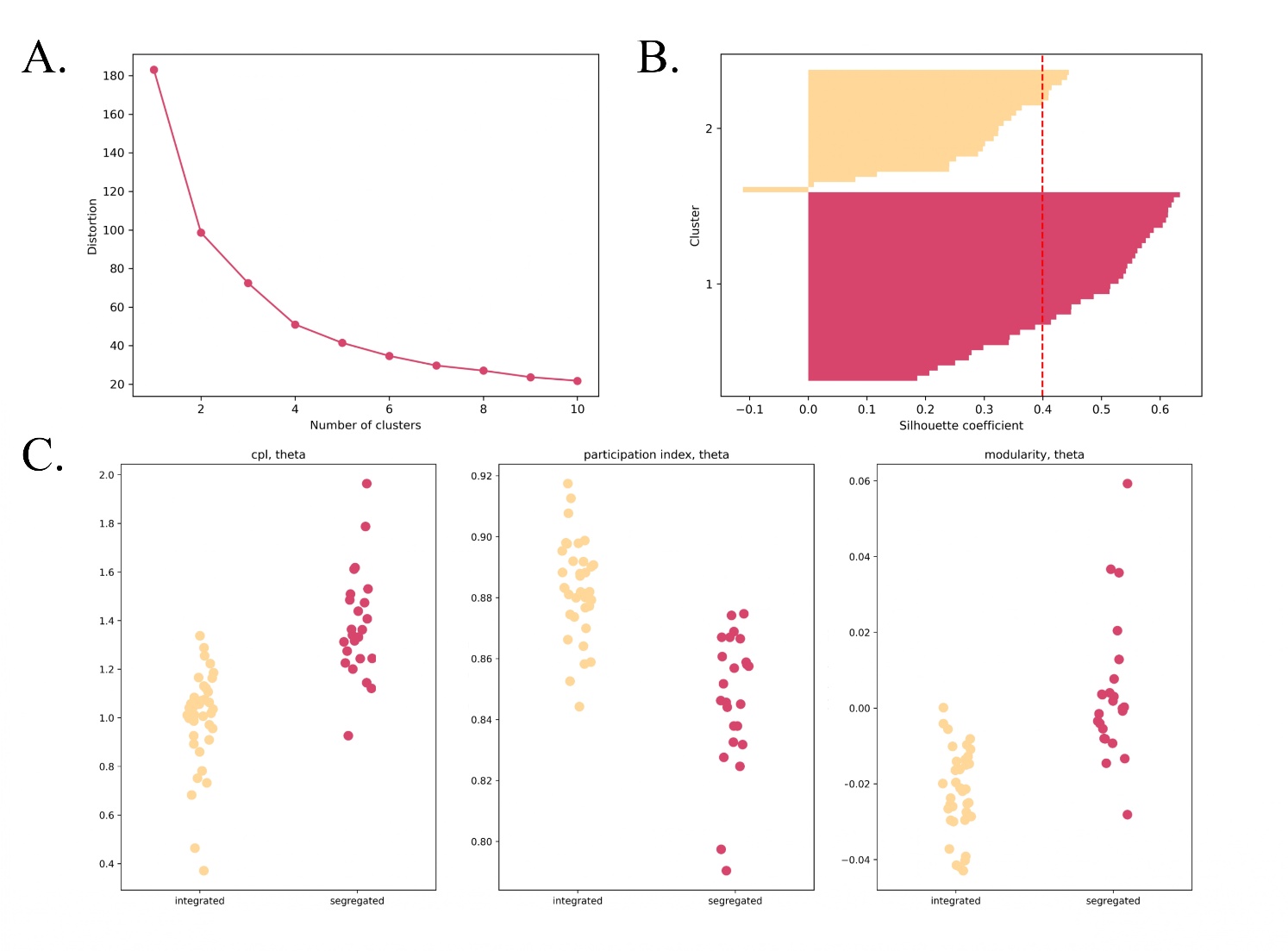


**Supplementary Figure 3.1.** Clustering quality for the **theta band, threshold 50%**. Panel A depicts distortion (elbow) plot. Panel B depicts silhouette diagram. A dashed line represents the mean silhouette coefficient. Panel C depicts pointplots for CPL (left), partial index (middle) and modularity (right) for 2 clusters highlighting between-cluster differences in the distribution of the variables.


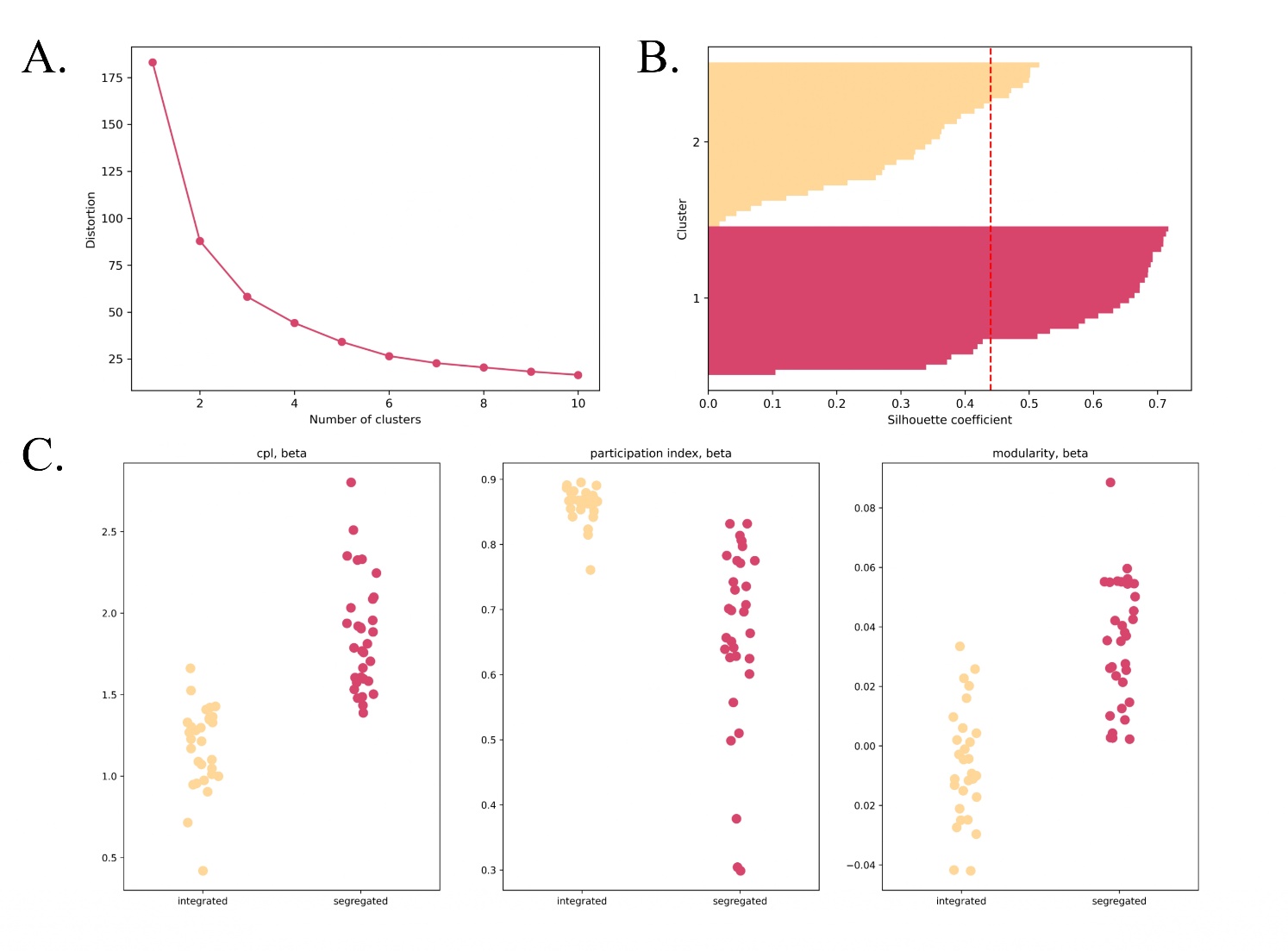


**Supplementary Figure 3.2.** Clustering quality for the **beta band, threshold 50%**. Panel A depicts distortion (elbow) plot. Panel B depicts silhouette diagram. A dashed line represents the mean silhouette coefficient. Panel C depicts pointplots for CPL (left), partial index (middle) and modularity (right) for 2 clusters highlighting between-cluster differences in the distribution of the variables.


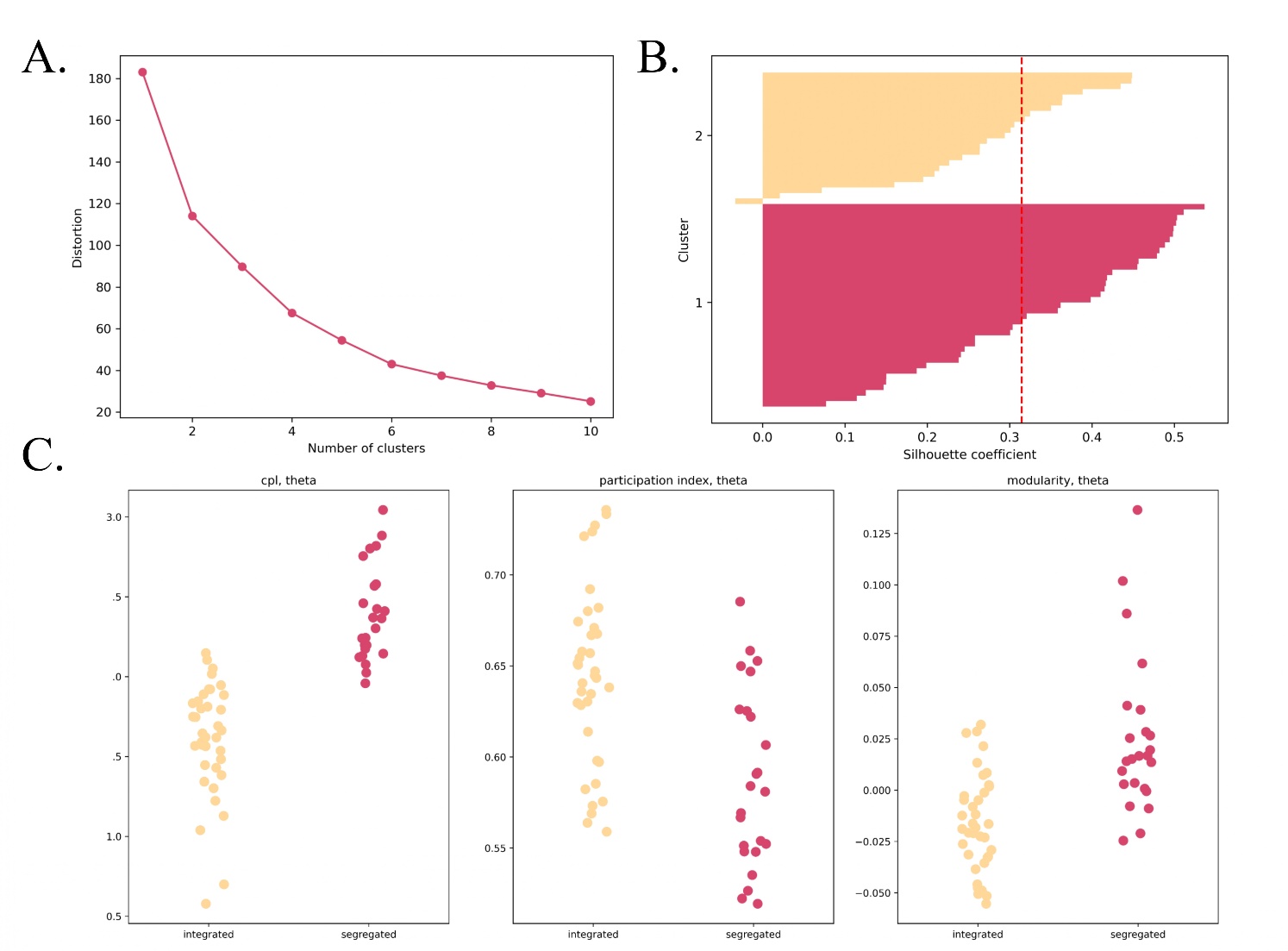


**Supplementary Figure 3.3.** Clustering quality for the **theta band, threshold 80%**. Panel A depicts distortion (elbow) plot. Panel B depicts silhouette diagram. A dashed line represents the mean silhouette coefficient. Panel C depicts pointplots for CPL (left), partial index (middle) and modularity (right) for 2 clusters highlighting between-cluster differences in the distribution of the variables.


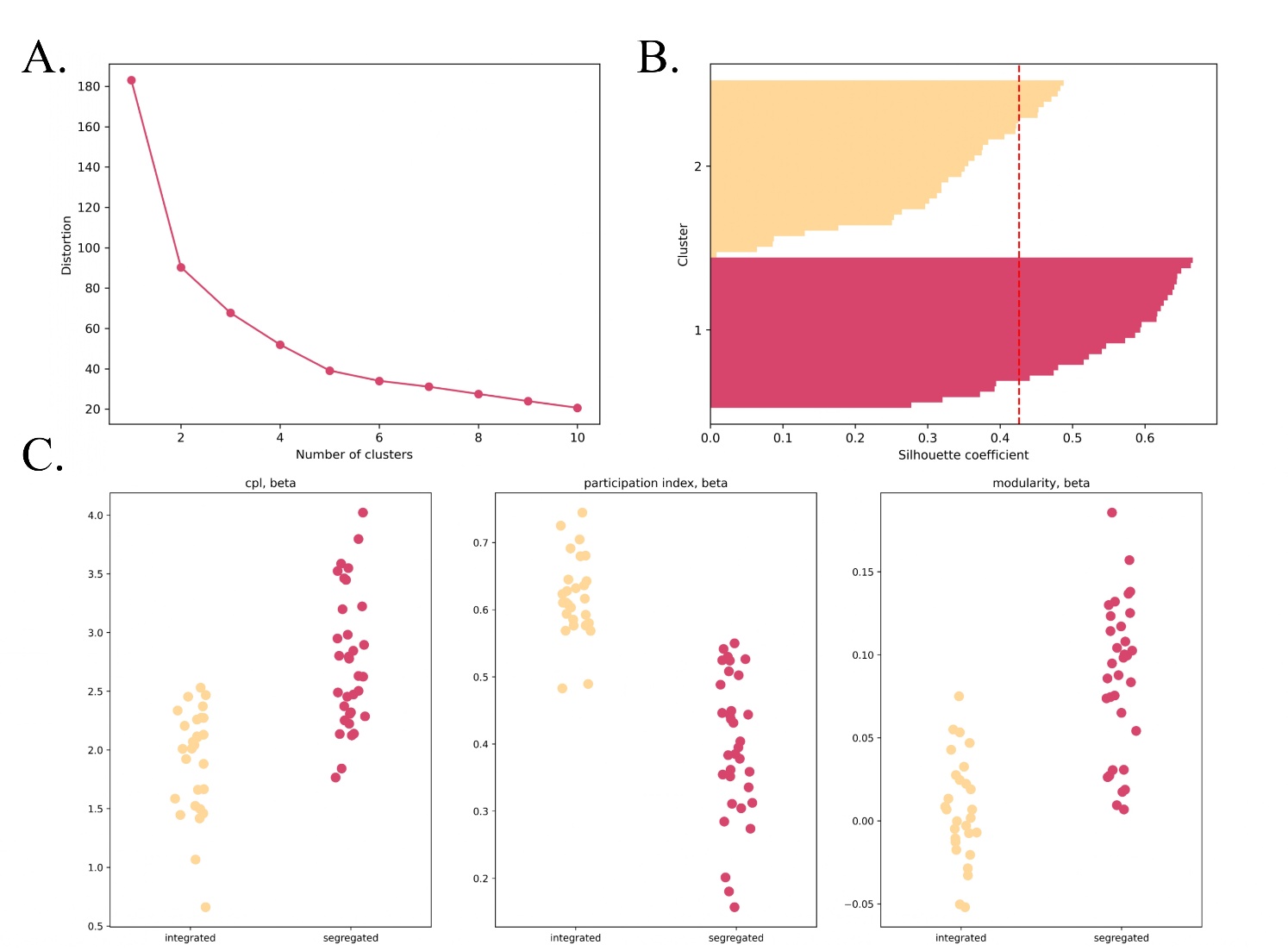


**Supplementary Figure 3.4.** Clustering quality for the **beta band, threshold 80%**. Panel A depicts distortion (elbow) plot. Panel B depicts silhouette diagram. A dashed line represents the mean silhouette coefficient. Panel C depicts pointplots for CPL (left), partial index (middle) and modularity (right) for 2 clusters highlighting between-cluster differences in the distribution of the variables.


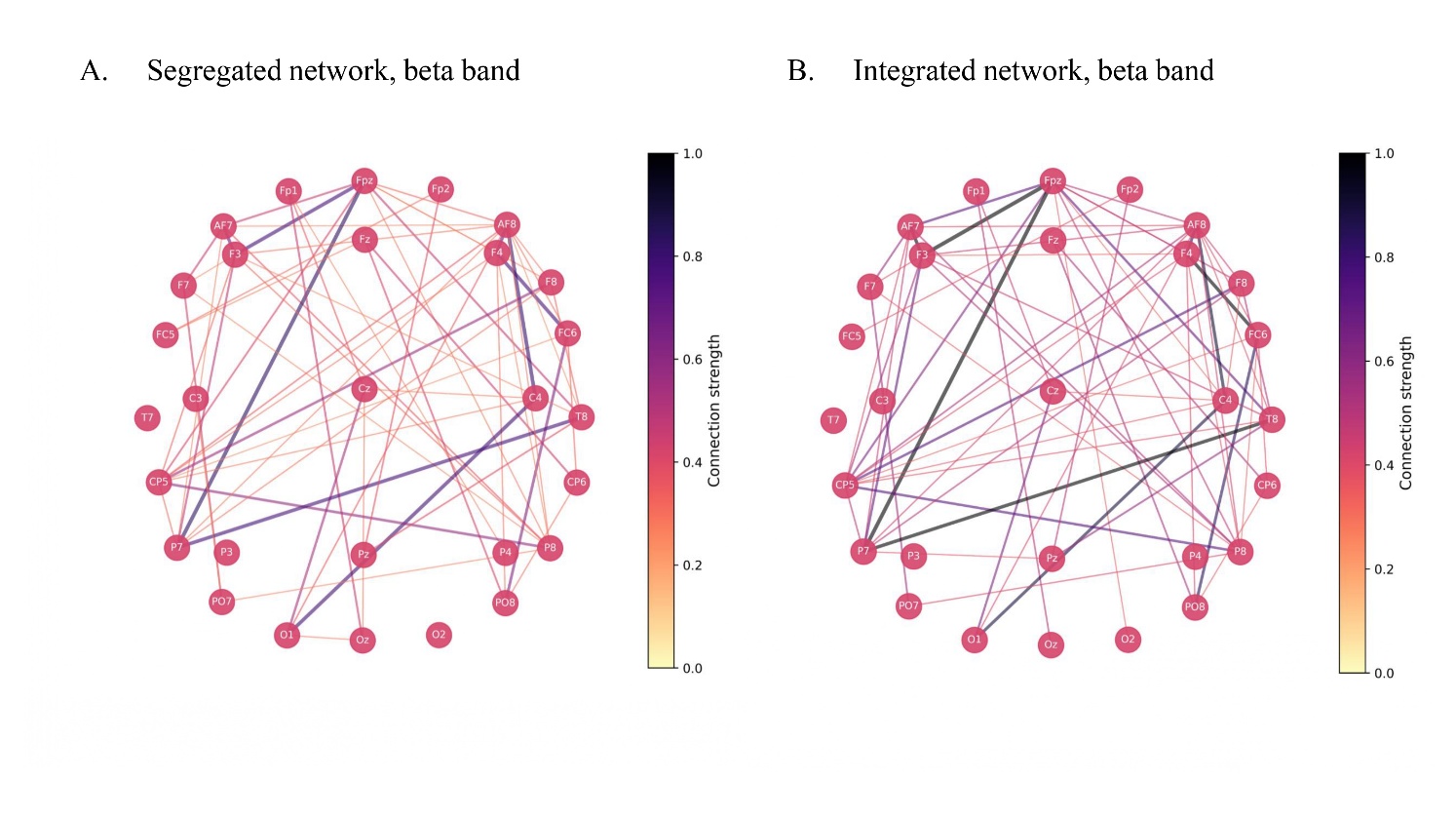


**Supplementary Figure 3.5.** The averaged brain network topology for children with integrated (left) and segregated (right) brain networks, beta band. The darker color indicates higher connectivity strength

# Supplementary Table 3.1. Between-cluster differences in CPL, modularity, participation index

|  |  | Integrated  M (SD) | Segregated  M (SD) | Mean difference | Manna-Whitney U stats | p-value |
| --- | --- | --- | --- | --- | --- | --- |
| Beta, 50%  N (int) = 31  N (seg) = 32 | CPL | 1.19 (0.25) | 1.86 (0.34) | -0.67 | 21.0 | <0.001** |
|  | Modularity | -0.05 (0.02) | 0.03 (0.02) | -0.04 | 68.0 | <0.001** |
|  | Participation index | 0.86 (0.03) | 0.67 (0.14) | 0.19 | 975.0 | < 0.001** |
| Theta, 50%  N (int) = 39  N (seg) = 24 | CPL | 1.00 (0.20) | 1.38 (0.22) | -0.39 | 63.0 | < 0.001** |
|  | Modularity | -0.02 (0.01) | 0.00 (0.02) | -0.03 | 66.0 | < 0.001** |
|  | Participation index | 0.88 (0.02) | 0.85 (0.02) | 0.03 | 878.0 | <0.001** |
| Beta, 80%  N (int) = 29  N (seg) = 34 | CPL | 1.93 (0.45) | 2.73 (0.59) | -0.80 | 133.0 | < 0.001** |
|  | Modularity | 0.008 (0.03) | 0.09 (0.05) | -0.08 | 83.0 | < 0.001** |
|  | Partial index | 0.62 (0.06) | 0.40 (0.11) | 0.22 | 969.0 | <0.001** |
| Theta, 80%  N (int) = 39  N (seg) = 24 | CPL | 1.62 (0.35) | 2.39 (0.30) | -0.77 | 12.0 | <0.001** |
|  | Modularity | -0.02 (0.02) | 0.03 (0.04) | -0.04 | 148.0 | <0.001** |
|  | Participation index | 0.65 (0.05) | 0.59 (0.05) | 0.06 | 746.0 | <0.001** |

*Note.* *p-value < 0.05; **p-value < 0.01. N = 63

# Association between clusters and CBT accuracy


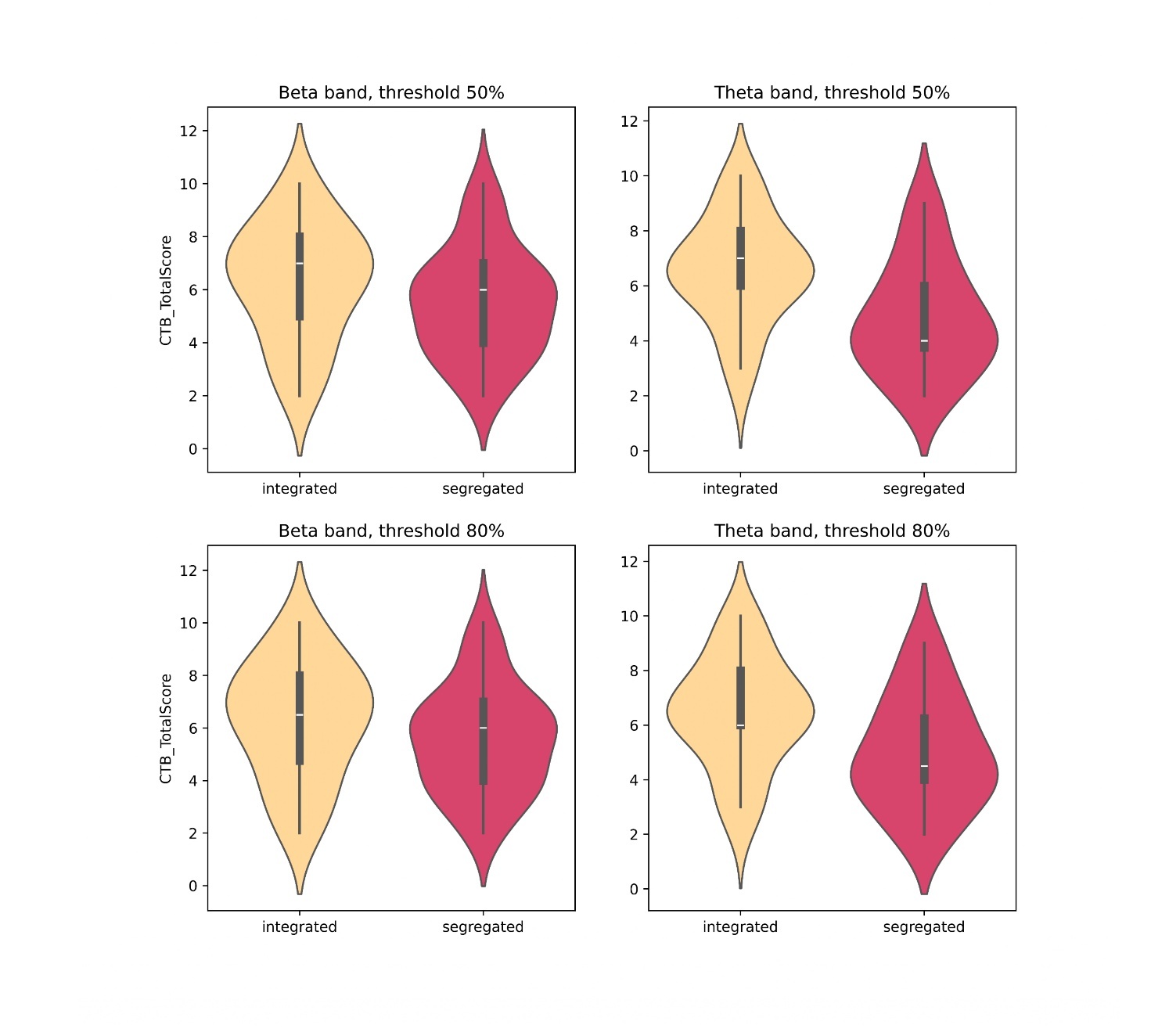


**Supplementary Figure 4.1.** Between-cluster differences in terms of CBT accuracy. Top left panel – beta band, threshold 50%; Top right panel – theta band, threshold 50%, Bottom left panel – beta band, threshold 80%; bottom right panel – theta band, threshold 80%

# Supplementary Table 4.1. Contingency table for clusters over beta and theta band, threshold 50%

|  | Integrated, theta | Segregated, theta |
| --- | --- | --- |
| Integrated, beta | 24 | 7 |
| Segregated, beta | 15 | 17 |

*Note.* N = 63

# Supplementary Table 4.2. Contingency table for clusters over beta and theta band, threshold 80%

|  | Integrated, theta | Segregated, theta |
| --- | --- | --- |
| Integrated, beta | 22 | 7 |
| Segregated, beta | 17 | 17 |

*Note.* N = 63


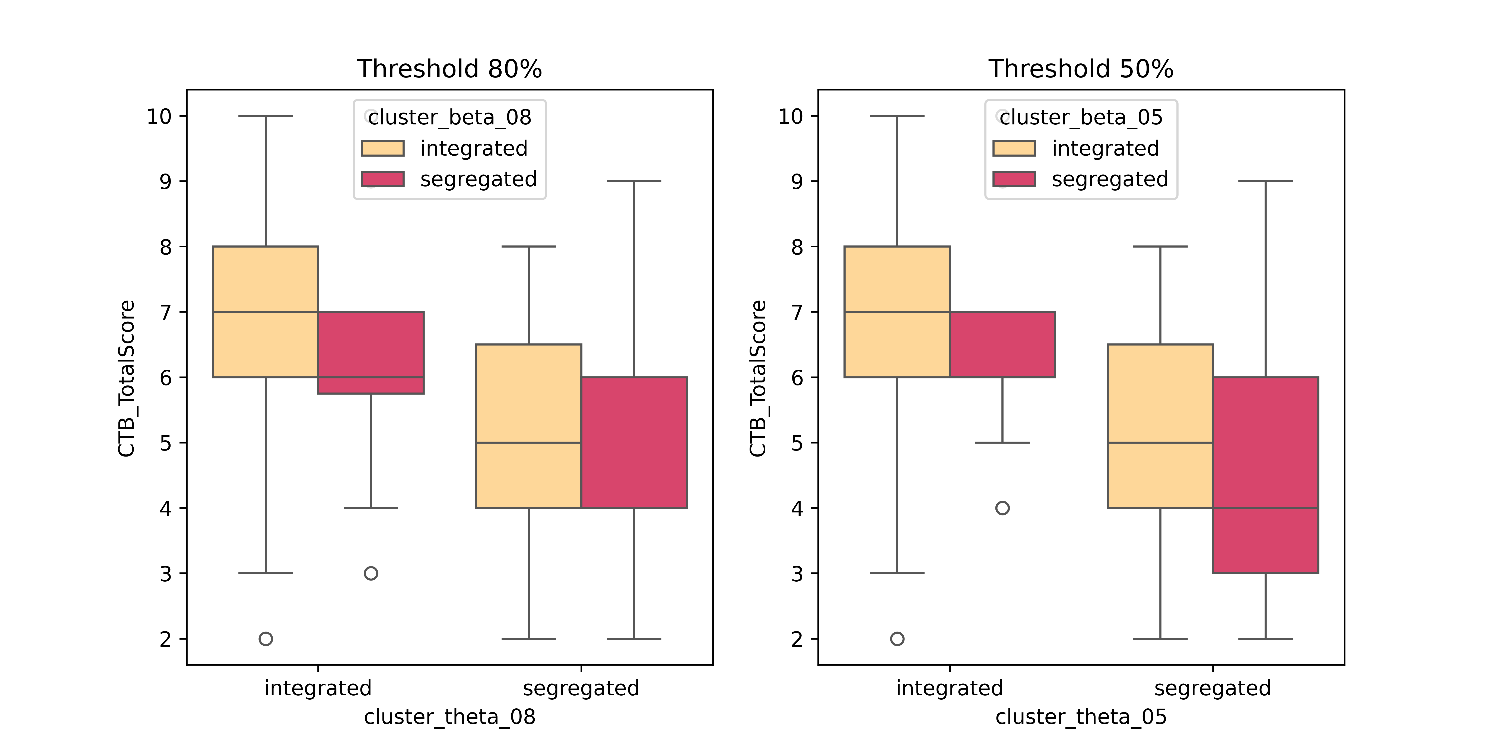


**Supplementary Figure 4.2.** The effect of clustering over beta band, while the effect of clustering over theta band is controlled for thresholds 50% (left panel) and 80% (right panel)

# Comparison of CPL, modularity and participation index in the theta and beta bands


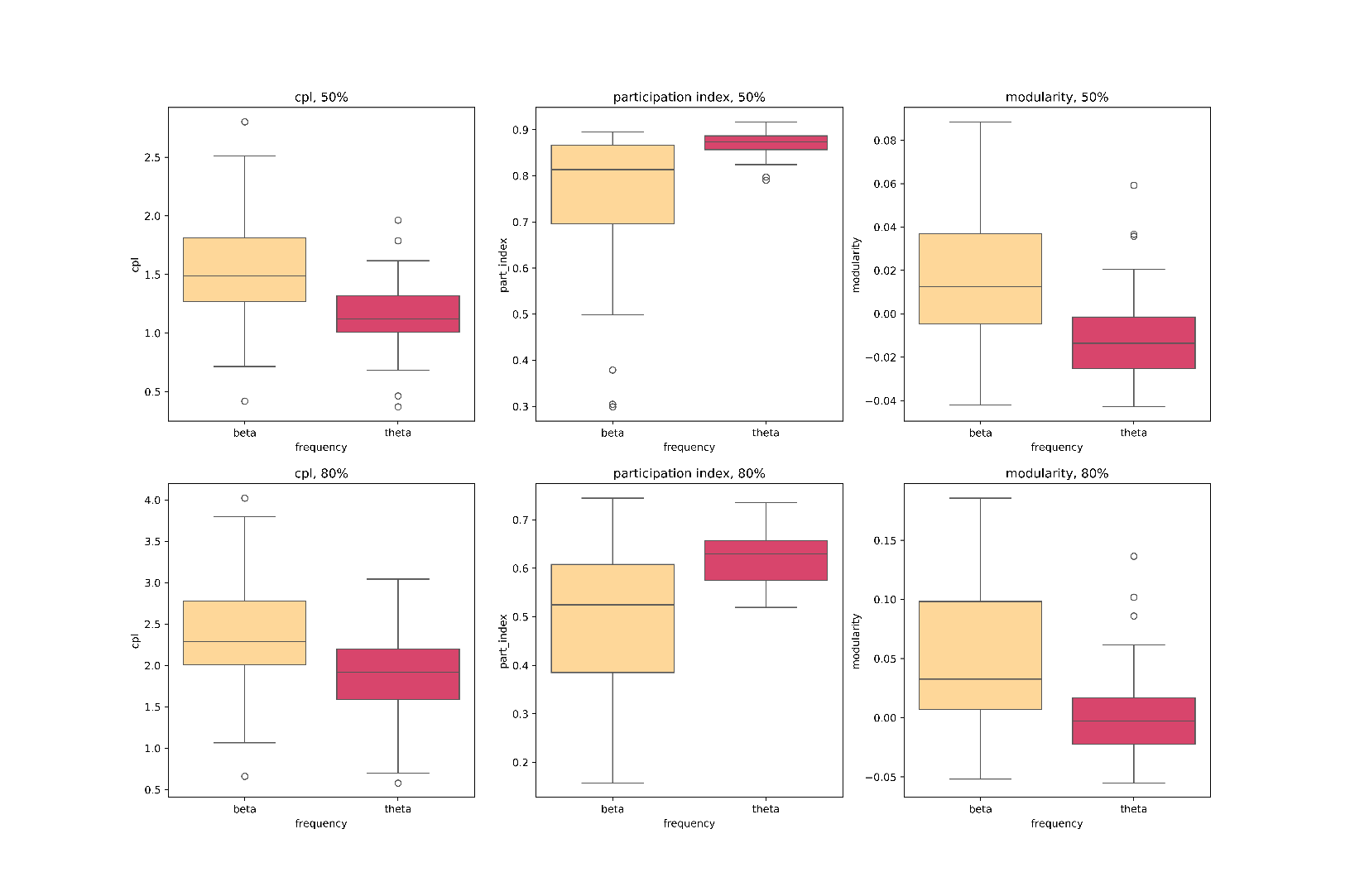


**Supplementary Figure 5.** Comparison of CPL, modularity and participation index in the theta and beta bands

# Supplementary Table 5.1. Comparison of CPL, modularity and participation coefficient distribution on theta and beta frequences, threshold 50%

|  | Beta mean (SD) | Theta mean (SD) | Mean difference | T-test for depended samples | P-value |
| --- | --- | --- | --- | --- | --- |
| CPL | 1.54 (0.46) | 1.15 (0.28) | 0.39 | 8.80 | <0.001** |
| Modularity | 0.02 (0.03) | -0.01 (0.02) | 0.03 | 8.46 | <0.001** |
| Participation coefficient | 0.76 (0.14) | 0.87 (0.03) | -0.11 | -6.40 | <0.001** |

*Note.* *p-value < 0.05; **p-value < 0.01. N = 63

# Supplementary Table 5.2. Comparison of CPL, modularity and participation coefficient distribution on theta and beta frequences, threshold 80%

|  | Beta mean (SD) | Theta mean (SD) | Mean difference | T-test for depended samples | P-value |
| --- | --- | --- | --- | --- | --- |
| CPL | 2.36 (0.67) | 1.92 (0.51) | 0.44 | 7.08 | <0.001** |
| Modularity | 0.05 (0.06) | 0.00 (0.04) | 0.05 | 7.83 | <0.001** |
| Participation coefficient | 0.50 (0.14) | 0.62 (0.06) | -0.12 | -8.30 | <0.001** |

*Note.* *p-value < 0.05; **p-value < 0.01. N = 63
